# Sustainable and Energy-Efficient Hydrogen Generation from NaBH_4_ Methanolysis Enabled by Nb_2_O_5_ Synthesized through a Pressureless Microwave Hydrothermal Method

**DOI:** 10.1021/acsomega.6c01184

**Published:** 2026-07-27

**Authors:** Fábio C. Riemke, Cristiane Raubach, Bruno Noremberg, Humberto V. Fajardo, Adilson C. da Silva, Dilean T. D. de Souza, Marcia T. Escote, Catherine Especel, Gwendoline Lafaye, Laurence Vivier, Antoninho Valentini, Murilo Salem, Neftali Lenin Villarreal Carreno

**Affiliations:** † Programa de Pós-Graduação em Ciência e Engenharia de Materiais, Centro de Desenvolvimento Tecnológico, Universidade Federal de Pelotas, Pelotas, RS 96010-000, Brasil; ‡ Departamento de Química, Instituto de Ciências Exatas e Biológicas, Universidade Federal de Ouro Preto, Campus Universitário Morro do Cruzeiro, Ouro Preto, MG 35402-136, Brasil; § Centro de Engenharia, Modelagem e Ciências Sociais Aplicadas (CECS), Universidade Federal do ABC (UFABC), São Paulo, SP 09606-045, Brasil; ∥ 27077 Université de Poitiers, CNRS, Institut de Chimie des Milieux et Matériaux de Poitiers-IC2MP, 4 Rue Michel Brunet, TSA 51106, Poitiers Cedex 9 86073, France; ⊥ Laboratório de Adsorção e Catálise, 308253 Universidade Federal do Ceará, Campus do Pici, Fortaleza, CE 60440-900, Brasil; # 37902 Centro de Dispositivos Embarcados e Pesquisa em Agricultura Digital SENAI-RS, São Leopoldo, RS 93025-753, Brasil

## Abstract

A pressureless microwave-assisted hydrothermal route is presented for the synthesis of highly active amorphous niobium pentoxide (Nb_2_O_5_·nH_2_O) nanostructures and their application as metal oxide catalysts for hydrogen generation via sodium borohydride methanolysis. The synthesis, conducted at sub-boiling temperature (75 °C) under open-vessel conditions, produces a microporous Nb_2_O_5_ material with a very high specific surface area (230 m^2^ g^–1^), abundant surface hydroxyl groups, and a predominance of weak-to-moderate acidic sites. Structural and surface analyses reveal a disordered NbO_6_ network enriched with terminal Nb=O species and undercoordinated oxygen environments, which are absent in crystalline commercial Nb_2_O_5_. In NaBH_4_ methanolysis, the amorphous Nb_2_O_5_ catalyst achieves a hydrogen generation rate of 22,781.7 mL min^–1^ g^–1^ at 25 °C with a low apparent activation energy of 21.95 kJ mol^–1^, outperforming many reported noble-metal-based catalysts. Reaction kinetics follow a first-order dependence on NaBH_4_ concentration, and the catalyst maintains stable activity over multiple reuse cycles. In addition, machine learning-assisted analysis was employed to process particle segmentation data and correlate morphological descriptors with catalytic performance, enabling quantitative assessment of particle size distributions, skewness, kurtosis, and aggregation behavior. These data-driven insights corroborate the experimentally observed structure–activity relationships, linking controlled nucleation under microwave irradiation to enhanced catalytic efficiency. The results establish amorphous Nb_2_O_5_ as a cost-effective and sustainable catalyst for on-demand hydrogen generation and highlight pressureless microwave synthesis combined with machine learning as a powerful framework for rational catalyst design.

## Graphical abstract

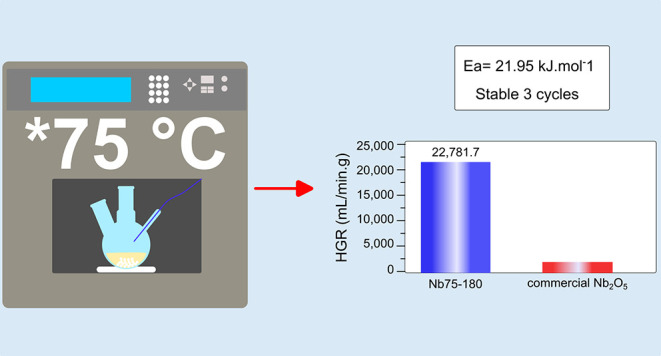

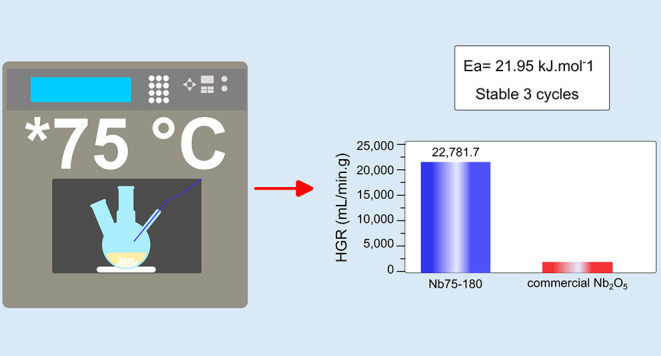

## 1 Introduction

Global concern involving environmental pollution imputes to hydrogen energy the status of a pivotal clean fuel source for the future.^1^ Nevertheless, its large-scale implementation faces some challenges related to its production, storage, distribution, and end-use efficiency.^2^ Perhaps storage is still the biggest question to be resolved.^3^ Hydrogen can be physically stored in pressurized vessels in the form of molecular hydrogen and in liquefied hydrogen tanks. However, this way can be unfavorable for practical purposes, as it requires complex engineering.^2–4^ To circumvent this obstacle, hydrogen storage in solid-state materials appears to be an alternative promising solution.

A lot of attention has been paid to chemical compounds, especially hydrides.^5^ Within this class, sodium borohydride (NaBH_4_) has been widely considered due to its large storage (stores nearly 10.8 wt % of hydrogen) and release capacities for hydrogen, as well as its nontoxic and nonflammable nature.^2,6,7^ The stored hydrogen can be released in several ways, including hydrolysis (eq 1) or methanolysis (eq 2).

1 $${\mathrm{NaBH}}_{4}+2H_{2}O\to {\mathrm{NaBO}}_{2}+4H_{2}$$

2 $${\mathrm{NaBH}}_{4}+4{\mathrm{CH}}_{3}\mathrm{OH}\to \mathrm{NaB}{\left({\mathrm{OCH}}_{3}\right)}_{4}+4H_{2}$$

Nonetheless, the use of methanol can bring some advantages as follows. (i) Faster kinetics, even at low temperatures, being possible to operate at subzero temperatures due to the low freezing point of methanol. (ii) When comparing the byproducts—NaBO_2_ and NaB­(OCH_3_)_4_—methanolysis presents a reduced issue regarding the deposition of byproducts on the catalyst surface (which can inhibit hydrogen production efficiency); this is because NaBO_2_ exhibits low solubility in water, whereas NaB­(OCH_3_)_4_ demonstrates significantly higher solubility in the reaction medium and, consequently, the latter is less prone to surface deposition, mitigating potential hindrances to catalytic activity and hydrogen yield. (iii) NaB­(OCH_3_)_4_ can be easily reconverted to the reactant. (iv) Methanol can be obtained from renewable sources which gives an environmentally friendly character to the process.^2,6^

Although methanolysis is thermodynamically spontaneous, the performance of this reaction must be enhanced with effective catalysts. Catalytic materials are broadly categorized into two classes: metal-containing catalysts (including noble and non-noble metals) and metal-free catalysts (e.g., carbon-based, silicon-based, and biobased materials). It is well established that noble-metal-based catalysts exhibit superior performance in methanolysis reactions. However, such catalysts exhibit significant drawbacks, including high production costs, limited durability, and a potential environmental impact. As a sustainable alternative, metal oxide catalysts (as opposed to supported metallic nanoparticles) have garnered increasing attention for their role in efficiently promoting methanolysis reactions.^8^

To the best of our knowledge, niobium pentoxide (Nb_2_O_5_) has not yet been explored as a catalyst for the methanolysis of sodium borohydride.^9–11^ Nb_2_O_5_ offers distinct advantages for catalytic applications, as it is readily synthesized, exhibits high chemical stability, is nontoxic, and is commercially available. Notably, Nb_2_O_5_ possesses diverse niobium coordination environments, including distorted NbO_6_ octahedra, NbO_4_ tetrahedra, and rarer polyhedral units such as NbO_5_, NbO_7_, and NbO_8_. These structural features contribute to its versatile catalytic behavior.^8–10^ In amorphous Nb_2_O_5_, the breakdown of long-range atomic connections results in undercoordinated oxygen atoms that act as drivers for surface hydroxylation as the material interacts with ambient moisture or aqueous environments.^11,12^ Under the synthesis conditions conducted here, Nb_2_O_5_ manifests as niobic acid (Nb_2_O_5_·nH_2_O). The presence of labile protons in niobic acid not only underscores its strong Brønsted acidity (H_0_ ≈ −5.6, comparable to 70% H_2_SO_4_ solution) but also highlights how these protons facilitate interactions with water molecules during dehydration processes.^13^ Emerging insights suggest that the highly polarized surface of Nb_2_O_5_ may enable it to retain its Lewis acidity even in the presence of water, drawing parallels with rare earth metal triflates, which are known for their robust Lewis acidity under similar conditions.^8^

In this context, microwave-assisted pressureless hydrothermal synthesis (MAPHS) emerges as a transformative approach, offering unique advantages that directly enhance the physicochemical properties of Nb_2_O_5_. One of the most notable benefits of MAPHS is its ability to accelerate the reaction kinetics through rapid and uniform volumetric heating. This mechanism significantly reduces synthesis times, often from hours or days to hours/mins, while ensuring consistent heat distribution throughout the reaction medium.^14,15^ Whereas many methods depend on external heat conduction, microwave heating operates from the inside outward, powered by two processes: the orientation of permanent or induced dipoles and the movement of an induced current.^16^ In addition to ensuring even heating, the process minimizes the formation of localized hot spots, which can otherwise lead to particle sintering or aggregation.^17^

Despite the benefits, conventional microwave hydrothermal synthesis remains shackled to expensive, high-pressure reactors—requiring harsh conditions just to surpass water’s boiling point. This work breaks free with open-vessel MAPHS, slashing costs and complexity by achieving superior nanocatalysts at ambient pressure, without sacrificing performance or control. This synthesis strategy constitutes a significant leap over conventional MAPHS approaches, as it enables the formation of highly active Nb_2_O_5_ catalysts under sub-boiling conditions and without the need for high-pressure autoclaves (a technical simplification that reduces energy consumption, enhances safety, and expands scalability). This method also aligns with green chemistry principles by minimizing energy input and eliminating high-pressure systems.

Moreover, to the best of our knowledge, no prior study has reported such an outstanding hydrogen generation performance from Nb_2_O_5_ synthesized under open-vessel, pressureless microwave conditions, underscoring the transformative potential of this route. The observed sub-boiling-point synthesis behavior can be attributed to a combination of nonthermal microwave effects, such as dipolar polarization and electric field interactions, along with potential localized heating from ionic species or catalytic hot spots, as thoroughly explained by Stuerga.^17^ In the case of Nb_2_O_5_, the rapid kinetics facilitate the straightforward synthesis of nanoparticles, reducing the need for annealing steps that might lead to drawbacks such as acidity depletion at higher pretreatment temperatures.^13^ The localized superheating induced by microwave irradiation facilitates its rapid nucleation and controlled growth, leading to the formation of usually smaller, more uniform nanoparticles with narrow size distributions.^14,18^

## 2 Experimental Part

### 2.1 Materials Synthesis

Nb_2_O_5_ sample, labeled Nb75-180, was synthesized by dissolving 14.8 mmol (4 g) of NbCl_5_ (CBMM) in 50 mL of distilled water under magnetic stirring. Upon the addition of 4 mL of 20% H_2_O_2_, the solution transitioned from a white, translucent suspension to a pale-yellow color. The mixture was placed into a borosilicate volumetric balloon featuring open cavities within an air atmosphere, inside a SEM Mars microwave. Temperature was monitored *in situ* via a fiber-optic thermocouple integrated into the volumetric balloon, with the microwave control system programmed to heat the solution at a ramp rate of 5 °C/min until reaching 75 °C. At this temperature, the system switched to continuous heating mode to maintain a thermal plateau for 180 min. The reaction yielded a biphasic mixture comprising a settled white powder and a supernatant pale-yellow liquid. The supernatant was discarded after centrifugation at 3500 rpm for 15 min. The residual white powder was then resuspended in distilled water and subjected to vortex agitation to remove water-soluble impurities from its porous structure. This purification process (centrifugation followed by redispersion) was repeated five times to ensure complete removal of contaminants. Finally, the purified powder was dried at 100 °C for 24 h in a convection oven to obtain the ceramic oxide material. For this study, a commercial Nb_2_O_5_ was kindly provided by CBMM (Companhia Brasileira de Metalurgia e Mineração).

### 2.2 Characterization Methods

#### 2.2.1 X-ray Diffraction (XRD)

The X-ray diffraction (XRD) analysis was conducted using a Bragg–Brentano geometry diffractometer (Shimadzu XRD-6000, Shimadzu Corporation, Japan) equipped with Cu–Kα radiation (λ = 1.5418 Å). The measurements were performed under operating conditions of 30 kV and 30 mA. The diffraction patterns were recorded over a 2θ range of 10° to 80°, employing a step size of 0.02° and a scanning rate of 2° min^–1^. Surface area and pore characteristics were analyzed via nitrogen adsorption–desorption isotherms at 77 K using a Micromeritics TriStar II Plus surface area analyzer.

#### 2.2.2 Surface Area Pore Size Distribution

The Brunauer–Emmett–Teller (BET) method was employed to calculate the specific surface area from the linear region of the adsorption isotherm (relative pressure *P*/*P* ^0^ = 0.05–0.30). Monolayer adsorption capacity was derived using the Langmuir model. Pore size distributions were determined using the Barrett–Joyner–Halenda (BJH) algorithm for mesopores (2–50 nm) and the t-plot method for micropores (<2 nm). Adsorption–desorption hysteresis loops and isotherm classifications (Type I–VI) were interpreted according to IUPAC guidelines. Data acquisition and analysis were performed without thermal correction to preserve raw adsorption–desorption behavior. Raman spectra were acquired using a Witec alpha300 Raman spectrometer equipped with a 532 nm (green) excitation laser.

#### 2.2.3 Fourier Transform Infrared Spectroscopy (FTIR)

The FTIR spectrum was recorded using a spectrometer operating in the range of 4000–400 cm^–1^, with a resolution of 4 cm^–1^ and an average of 32 scans to enhance the signal-to-noise ratio. The sample was prepared by mixing it with potassium bromide (KBr) in a 1:100 mass ratio, followed by pellet pressing to ensure optimal infrared transparency.

#### 2.2.4 Scanning Electron Microscopy (SEM)

Scanning electron microscopy (SEM) images were captured with a JEOL-7500F microscope operating at an acceleration voltage of 2 kV.

#### 2.2.5 X-ray Photoelectron Spectroscopy (XPS)

X-ray photoelectron spectroscopy (XPS) was conducted using an Omicron analyzer (Concentric Hemispherical Analyzer—CHA), employing Al/Kα radiation with an energy of 1486.6 eV. The sample was deposited on copper tape mounted on molybdenum sample holders and transferred under atmospheric conditions to the prechamber. The chamber was operated at 1 × 10^–9^ mbar. The survey spectrum was obtained with a pass energy of 50 eV and a step size of 1 eV, while specific regions of interest were recorded with higher resolution, at a pass energy of 10 eV and a step size of 0.1 eV. The XPS binding energy calibration was performed using the C 1s peak at 284.8 eV as a reference. Quantitative analysis (atomic percentages and Nb:O ratios) was performed exclusively from the integrated areas of the high-resolution Nb 3d and O 1s regions rather than from the survey spectrum, using Shirley background subtraction and Scofield sensitivity factors; the survey spectrum was used solely for qualitative elemental identification.

#### 2.2.6 Pyridine Temperature-Programmed Desorption (TPD)

The sample (60 mg) was previously activated by heating at 160 °C under He flow (25 mL/min) for 1 h. After activation, the sample was cooled to 40 °C, and then pyridine vapor was introduced into the He flow for adsorption. Afterward, the sample was maintained at 50 °C under He flow until a constant baseline was reached. Subsequently, pyridine desorption was carried out up to 600 °C at 10 °C/min, and the gas composition was monitored using a thermal conductivity detector (TCD).

#### 2.2.7 Automated Particle Analysis Software

Quantitative morphological analysis of niobium particles was performed using a custom Python-based platform (Streamlit v1.32.0), integrating Cellpose deep learning segmentation with classical image processing for automated characterization of SEM micrographs. Spatial calibration was achieved either through automated extraction of pixel size metadata from microscopy .hdr files or manual scale bar measurement via an interactive graphical interface supporting multiple units (μm, nm, mm) with automatic conversion. The preprocessing pipeline (OpenCV v4.8.0) comprised: (i) 8-bit min-max normalization; (ii) CLAHE (clip limit: 0.0–10.0, default 1.7; tile size: 8 × 8 pixels); (iii) automatic polarity inversion when mean intensity >0.5; (iv) gamma correction (γ: 0.1–3.0, default 0.5) with a 1.15× multiplicative factor, clamped to [0,1]; (v) optional Gaussian blur (kernel: 0–10 pixels, default 0); and (vi) symmetric padding with a gray background (29/255) at 0–200% of image dimensions to reduce edge artifacts. Particle segmentation employed Cellpose v2.2.3 pretrained ‘cyto3’ model with GPU acceleration (PyTorch v2.0+). Operational parameters: flow_threshold = 0.0–4.0 (default 1.0), cellprob_threshold = −6.0 to 6.0 (default −50.0), and minimum particle size = 10 pixels. No model fine-tuning was performed to maintain reproducibility. Morphometric parameters extracted via scikit-image v0.21.0 regionprops included area (μm^2^), perimeter (μm), circularity (4π·area/perimeter^2^), aspect ratio (major/minor axis), solidity, eccentricity, orientation, and centroid coordinates. Quality control removed edge-touching particles and nonfinite values (NaN, ±∞). Dimensional constraints: minimum detectable area determined by the segmentation threshold and a maximum area limited by image dimensions. Statistical visualization (matplotlib v3.7.0, seaborn v0.12.0) generated a six-panel dashboard: (i) size distribution histograms (≤30 bins) with mean/median markers; (ii) circularity-area scatter plots with aspect ratio color mapping; (iii) diameter–aspect ratio phase diagrams; (iv) adaptive-binned size distribution pie charts; (v) multimetric boxplots; and (vi) descriptive statistics (mean ± SD). Adaptive bin ranges: <1 μm^2^ [0, 0.1, 0.2, 0.5, 1.0]; 1–5 μm^2^ [0, 0.5, 1.0, 2.0, 5.0]; and >5 μm^2^ [0, 1.0, 2.0, 5.0, 10.0, 20.0]. Particle-level data were exported in CSV format. Operational parameters included a flow threshold of 1.0 and a highly permissive cell probability threshold of −4.0 to maximize detection sensitivity. Inference was performed with test-time augmentation enabled (averaging predictions from 8 image rotations/flips) to enhance robustness. Following automated segmentation, mask artifacts could be manually refined using an interactive visual editor (deletion, merging, and creation of particles).

### 2.3 Hydrogen Generation Experiment

In a typical experiment, a certain mass of sodium borohydride (NaBH_4_, 97% Nuclear) was introduced into the reactor, which was kept inside a thermostatic water bath to maintain the reaction temperature controlled. Subsequently, an appropriate amount of the catalyst was loaded into the reactor. In the next step, 20 mL of methanol (Neon P. A.) was immediately added to the reactor. The hydrogen produced from the sodium borohydride methanolysis reaction was quantified using the classical water volume displacement technique. A graduated glass buret filled with water was connected to the top outlet of the reactor. The generated hydrogen volume was measured from the change of the water level in the buret. The cumulative hydrogen volume was read at certain times and represented graphically. The effects of catalyst mass, NaBH_4_ amount, CH_3_OH volume, and temperature on the hydrogen production rate were studied. The hydrogen generation rate (HGR) (mL of H_2_ min^–1^ g^–1^) and reaction rate (mL of H_2_ min^–1^) values were calculated according to the linear part of the volume of hydrogen amount versus time graphs, before reaching the plateau indicating the end of hydrogen production. The apparent activation energy of the reaction was determined according to Arrhenius law. In the Arrhenius equation (ln k = −E_a_/R­(1/T) + ln A), k is the rate constant, E_a_ is the activation energy of the reaction, R is the gas constant (8.314 J mol^–1^ K^–1^), T is the absolute temperature in Kelvin, and A is the frequency factor (Arrhenius constant). The recycling tests were carried out three times under specific conditions. After each reaction cycle, the catalyst was separated from the reaction medium via centrifugation, washed with distilled water, dried at 80 °C overnight, reweighed, and finally used for the next reaction cycle. Replication tests carried out on the catalytic unit used showed that the average error in quantifying hydrogen production did not exceed 2%.

## 3 Results and Discussion

### 3.1 X-ray Diffraction (XRD)

The X-ray diffraction (XRD) pattern of the Nb75-180 sample, as demonstrated in Figure 1, exhibits characteristic features indicative of an amorphous phase, with the absence of well-defined Bragg reflections and the presence of two broad diffraction halos.^19^ These halos arise from the short-range structural order inherent to the material and suggest a lack of periodicity beyond the nanometric scale.^20^ The intensity of the scattered X-rays decays to nearly zero at approximately 40° in 2θ, marking the limit of the diffuse scattering region. The amorphous nature of Nb_2_O_5_·nH_2_O is primarily associated with the structural disorder within the NbO_6_ octahedra. Unlike crystalline Nb_2_O_5_ polymorphs, where NbO_6_ units form well-ordered networks, the hydrated amorphous phase exhibits significant distortions due to a high density of point defects, including oxygen vacancies and hydroxyl terminations.^21^

**Figure 1:**
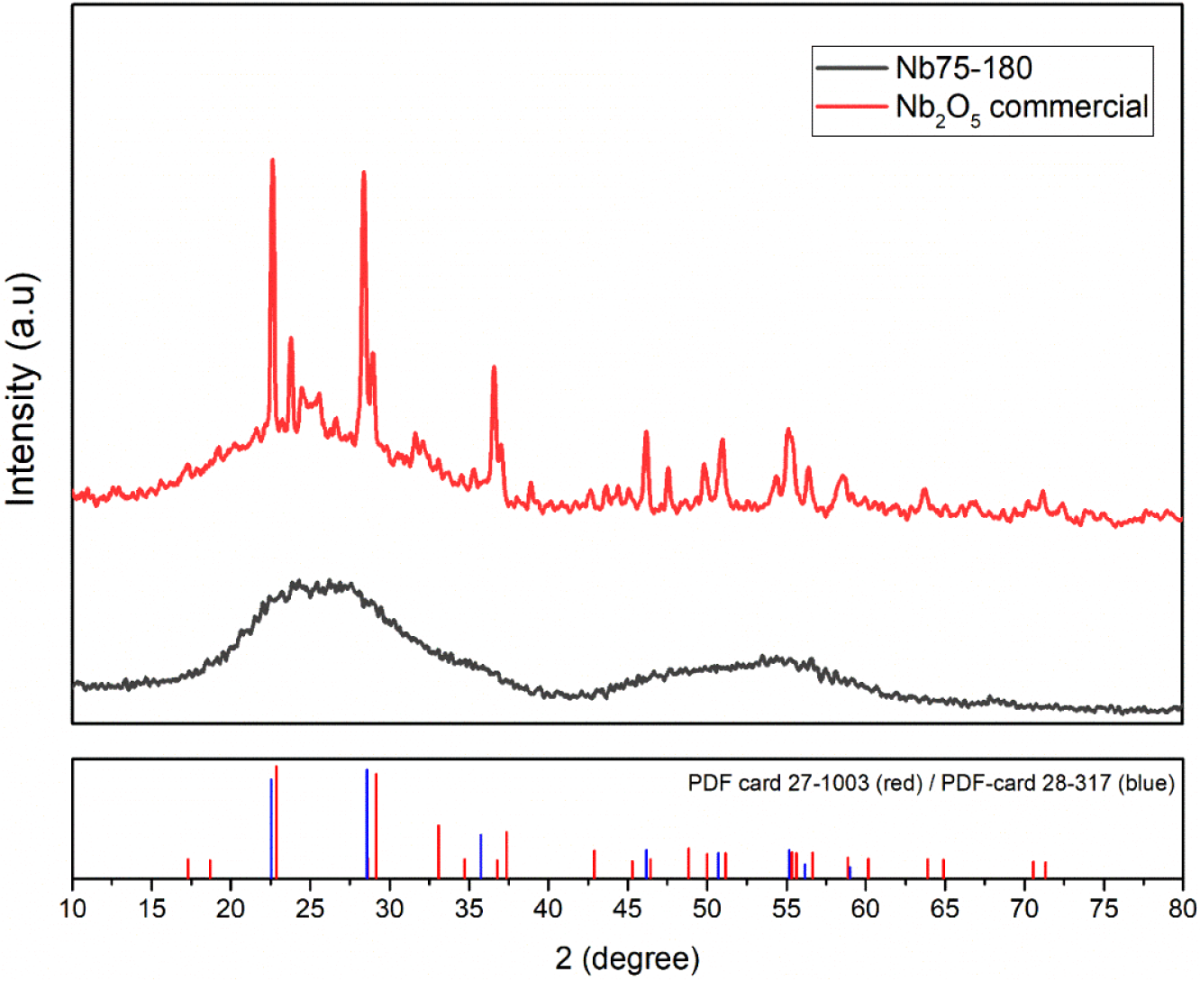
XRD patterns showing amorphous Nb_2_O_5_·nH_2_O (Nb75-180) with characteristic broad halos from disordered NbO_6_ octahedra and biphasic crystalline commercial Nb_2_O_5_ containing coexisting TT and T polymorphs.

These defects disrupt long-range periodicity and introduce local variations in Nb–O bond lengths and O–Nb–O angles, which collectively contribute to the broad diffraction features observed in the XRD pattern. The two broad halos represent a characteristic structural fingerprint of the short-range order within the disordered NbO_6_ network, arising from the random arrangement of nanocrystalline domains. These features reflect the presence of local coordination environments (primarily distorted NbO_6_ octahedra) that lack long-range periodicity. The resulting profile aligns well with previous reports on amorphous Nb_2_O_5_·nH_2_O, whose XRD signatures—marked by diffuse halos and the absence of sharp reflections—are inherently incompatible with standard Powder Diffraction File (PDF) entries based on crystalline counterparts. In contrast, the XRD pattern of the commercial Nb_2_O_5_ sample reveals a crystalline profile characterized by the coexistence of two distinct polymorphic phases. The first phase, formed at lower calcination temperatures, is identified by the presence of pseudohexagonal structural motifs, consistent with PDF card 27-1003 (TT-Nb_2_O_5_). The second phase exhibits an orthorhombic lattice arrangement, as corroborated by PDF card 28-317 (T-Nb_2_O_5_). The simultaneous occurrence of these two phases suggests that the thermal treatment conditions (specifically, the interplay between temperature and annealing time) resided in an intermediate regime. This regime was neither sufficiently mild to stabilize exclusively the low-temperature pseudohexagonal phase (typically formed at 500–550 °C) nor energetically vigorous enough to promote complete transformation into the high-temperature orthorhombic matrix (typically achieved at 700–750 °C). Consequently, the material retained a biphasic structure, reflecting an incomplete polymorphic transition arrested at intermediate thermal conditions.

### 3.2 Surface Area Pore Size Distribution

Figure 2a shows the N_2_ adsorption/desorption isotherms. The synthesized Nb_2_O_5_·nH_2_O sample exhibited an isotherm profile characteristic of Type 1­(b), which has a predominance of wider micropores.^22^ The synthesis method presented herein yielded a material with a specific surface area approximately 8-fold larger than that of the commercial sample.

**Figure 2:**
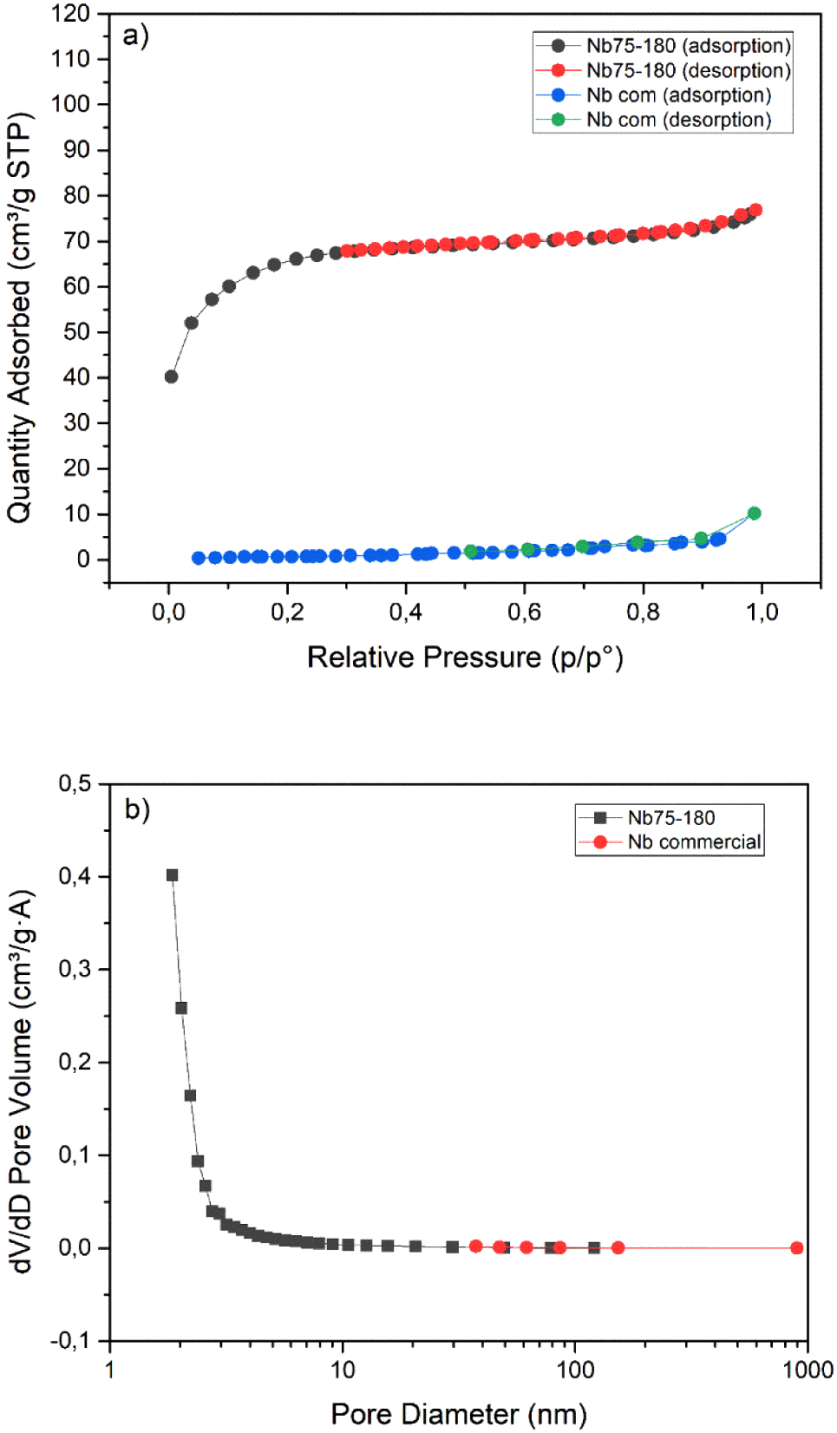
N_2_ adsorption/desorption isotherms (a) and pore size distributions (b) showing microporous Nb_2_O_5_·nH_2_O (Type 1b isotherm, BET area 230 m^2^/g) with predominant 2–3 nm pores versus nonporous commercial Nb_2_O_5_ (Type III isotherm, BET area 28 m^2^/g).

This classification is consistent with the high micropore contribution to the total surface area, which was quantified at 138 m^2^/g (t-plot analysis), while the Brunauer–Emmett–Teller (BET) surface area was 230 m^2^/g. The pore diameter distribution (Figure 2b), obtained using the Barrett–Joyner–Halenda (BJH) method, confirms the predominance of microporosity, with a contribution from pores with diameters between 2 and 3 nm. On the other hand, the commercial Nb_2_O_5_ sample shows a Type III isotherm with no hysteresis loop and no identifiable monolayer formation, consistent with nonporous or macroporous materials.^22^ The pore diameter distribution (Figure 2b) and the low BET surface area of 28 m^2^/g reveal the isotherm.

### 3.3 Raman Scattering Spectroscopy

The Raman spectrum for the samples exhibits characteristic features consistent with their known structural attributes as demonstrated in Figure 3a,b.

**Figure 3:**
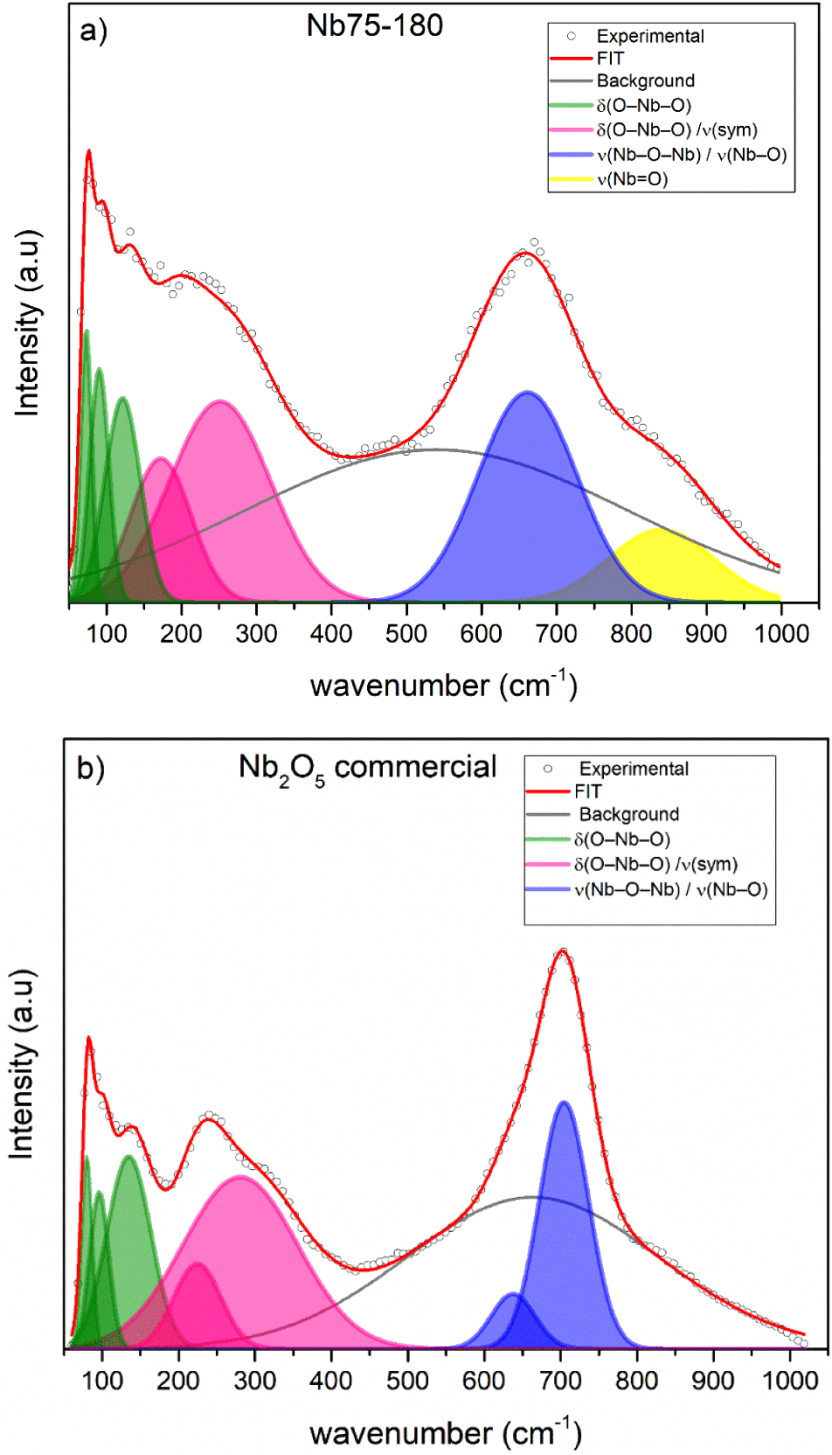
Baseline-corrected Raman spectra with Gaussian peak fitting for (a) amorphous Nb_2_O_5_·nH_2_O (Nb75-180) and (b) crystalline commercial Nb_2_O_5_, showing low-frequency lattice modes (<200 cm^–1^), Nb–O bending/stretching vibrations (252–280 cm^–1^), and high-frequency Nb–O–Nb stretching (637–661 cm^–1^). An exclusive terminal Nb=O mode at 845 cm^–1^ is observed in (a) but is absent in (b).

The modes, all below 200 cm^–1^, are typically associated with lattice vibrations or external modes in metal oxides. In niobium pentoxide and its hydrates, such low-frequency modes often correspond to translational and librational motions of the NbO_6_ octahedral units or collective vibrations of the framework influenced by hydrogen bonding from water molecules.^23^ Specifically, the peaks at 72 cm^–1^, 90 cm^–1^ (Nb75-180) and 80 cm^–1^, 96 cm^–1^ (commercial Nb_2_O_5_) may be attributed to lattice modes involving the displacement of niobium atoms within the oxygen framework, potentially modulated by the presence of H_2_O.^24^ The slightly higher frequencies at 122 cm^–1^ and 172 cm^–1^ (Nb75-180) and 135 cm^–1^ (commercial Nb_2_O_5_) could indicate librational modes of water molecules loosely bound to the Nb_2_O_5_ structure or bending vibrations of Nb–O–Nb bridges δ­(O–Nb–O). These assignments align with the general observation that hydration introduces additional low-frequency features due to the interaction of H_2_O with the oxide lattice. The peak at 252 cm^–1^ (Nb75-180) and 280 cm^–1^ (commercial Nb_2_O_5_) lies in a region commonly associated with bending or stretching vibrations of Nb–O bonds within the octahedral NbO_6_ units. In anhydrous Nb_2_O_5_, modes around 200–300 cm^–1^ are often assigned to O–Nb–O bending vibrations—δ­(O–Nb–O)—or symmetric stretching—νs/v­(sym)—of terminal Nb–O bonds in distorted polyhedra. The presence of water in Nb_2_O_5_ matrix is able to shift these modes slightly due to hydrogen bonding or structural disorder as demonstrated by the 28 wavenumber difference in δ­(O–Nb–O) between the sample Nb75-180 (Nb_2_O_5_·nH_2_O) and the (commercial Nb_2_O_5_).^25^ The prominent peaks at 661 cm^–1^ (Nb75-180) and 637 cm^–1^ (commercial Nb_2_O_5_) occur in the high-frequency region, characteristic of stretching vibrations of Nb–O bonds in niobium oxides—ν­(Nb–O–Nb)/ν­(Nb–O), respectively.^15^ The significantly larger peak area observed at 661 cm^–1^ in the amorphous hydrated phase compared to 637 cm^–1^ in the crystalline commercial Nb_2_O_5_ seems to be related to the relaxation of Raman selection rules and the overlap of multiple vibrational modes originating from the broad distribution of distorted NbO_6_ coordination environments present in the disordered structure. A distinct Raman mode at 845 cm^–1^ was observed exclusively in the Nb75-180 (Nb_2_O_5_·nH_2_O) spectrum, assigned to the stretching vibration of terminal Nb=O bonds (niobyl groups), which arise from undercoordinated niobium sites at the surface or within the amorphous network where the lack of long-range structural order permits the formation of nonbridging oxygen species that are largely absent in the fully condensed corner- and edge-sharing NbO_6_ polyhedral framework of the crystalline orthorhombic phase.

### 3.4 Fourier Transform Infrared Spectroscopy (FTIR)

The FTIR analysis for the samples is presented in Figure 4. For both samples, a broad, low-intensity absorption envelope spanning 500–750 cm^–1^ is attributed to Nb–O lattice phonon modes, reflecting structural vibrations within the niobium oxide framework. A distinct, narrow resonance at 878 cm^–1^ corresponds to the ν_as_(Nb=O), indicative of terminal oxo groups in a distorted octahedral coordination environment. The commercial Nb_2_O_5_ exhibits a markedly attenuated intensity in the hydroxyl stretching band, accompanied by a substantially reduced integrated area for the δ­(H–O–H) bending vibration at 1625 cm^–1^, relative to the Nb75-180 sample. This pronounced decrease in both ν­(O–H) and δ­(H–O–H) vibrational features indicates a lower concentration of surface-bound water molecules and hydroxyl groups in the commercial material. The diminished hydroxyl population is consistent with the crystalline nature of the commercial Nb_2_O_5_, where thermal treatment during synthesis promotes dehydroxylation and condensation of surface OH groups. Conversely, the enhanced intensity and broadening of these bands in Nb75-180 corroborate its amorphous, hydrated character (Nb_2_O_5_·nH_2_O), wherein structural water and terminal hydroxyl species are retained within the disordered NbO_6_ network.

**Figure 4:**
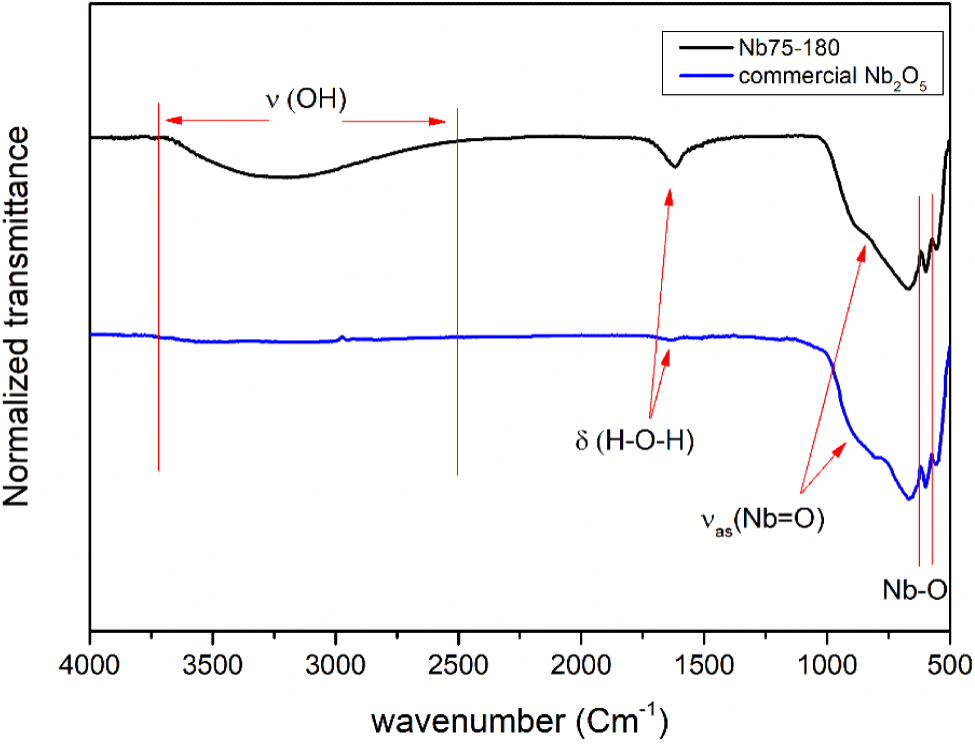
FTIR spectra showing Nb–O lattice modes (500–750 cm^–1^), terminal Nb=O stretching at 878 cm^–1^, and significantly enhanced O–H stretching and H–O–H bending (1625 cm^–1^) in hydrated amorphous Nb_2_O_5_·nH_2_O (Nb75-180) compared to dehydroxylated crystalline commercial Nb_2_O_5_.

### 3.5 Temperature-Programmed Desorption of Pyridine (TPD-Pyridine)

Figure 5 shows the pyridine TPD profiles for the Nb-75-180 and commercial Nb_2_O_5_ samples. The TPD experiments do not make it possible to distinguish between Brönsted and Lewis acid sites; however, it is possible to observe or indicate the presence of sites with various acid strengths. Therefore, one can assume the temperature ranges (up to 200, 200–400, and 400–600 °C) as weak, moderate, and strong acid sites, respectively. By assuming such classification, the commercial sample shows the predominance of moderate and strong acid sites, while the Nb75-180 sample shows the predominance of weak and moderate acid sites. The total amount of pyridine desorption for the Nb75-180 sample (122 μmol/g) was nearly five times higher than the amount determined for the commercial Nb_2_O_5_ sample (26 μmol/g).

**Figure 5:**
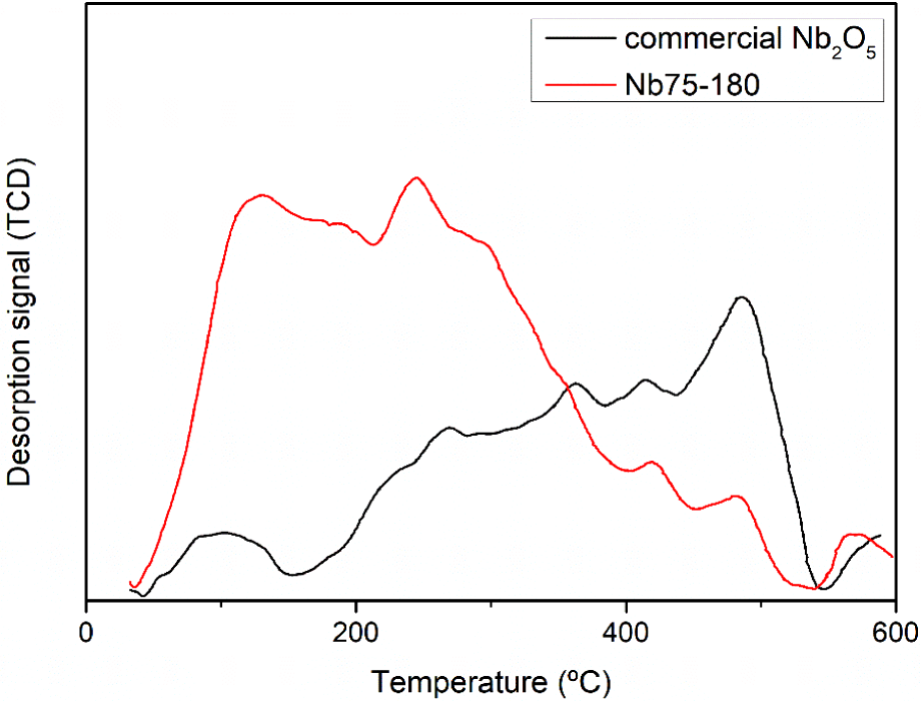
Temperature-programmed desorption of pyridine showing predominant weak and moderate acid sites in Nb75-180 (122 μmol/g total desorption) versus predominant moderate and strong acid sites in commercial Nb_2_O_5_ (26 μmol/g), with the amorphous sample exhibiting approximately 5-fold higher total acidity.

### 3.6 X-ray Photoelectron Spectroscopy (XPS)

The survey spectrum recorded during XPS analysis, as demonstrated in Figure 6, shows that only carbon, oxygen, and niobium can be detected at the surface of the Nb75-180 and the Nb_2_O_5_ commercial sample. The survey spectrum is used here solely for qualitative elemental identification; all subsequent interpretation of surface chemistry is based on the high-resolution Nb 3d and O 1s regions acquired at a pass energy of 10 eV, which resolve the individual chemical states with the precision required for the present discussion.

**Figure 6:**
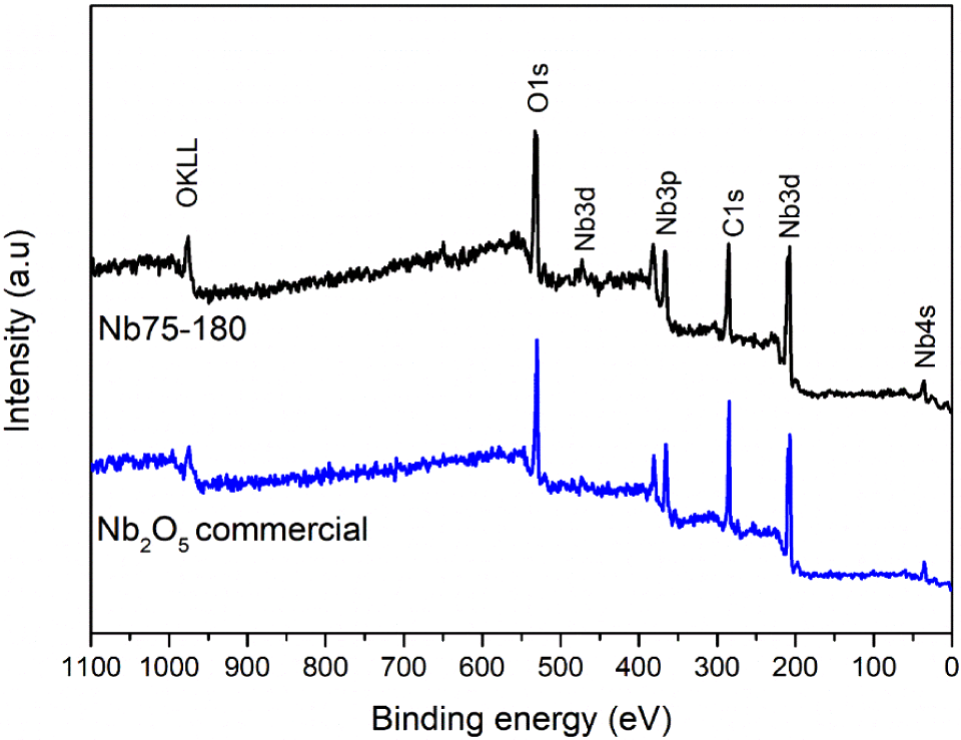
XPS survey spectra confirming the presence of only carbon, oxygen, and niobium at the surface of both Nb75-180 and commercial Nb_2_O_5_ samples.

The high-resolution Nb 3d spectra for the Nb75-180 sample presented in Figure 7a exhibit characteristic doublet peaks at 209.8 and 207.1 eV, attributed to the Nb 3d_3/2_ and Nb 3d_5/2_ spin–orbit components, respectively. These binding energies are consistent with those observed for the commercial Nb_2_O_5_ reference material, where the primary Nb 3d_3/2_(a) and Nb 3d_5/2_(a) components were centered at 209.77 and 207.08 eV, respectively. Additionally, the commercial Nb_2_O_5_ spectrum presented in Figure 7c reveals a minor secondary doublet at 208.22 and 205.12 eV (Nb 3d_3/2_(b) and Nb 3d_5/2_(b)). The ∼2 eV downshift relative to the main Nb^5+^ doublet is consistent with a small population of substoichiometric niobium sites (Nb_2_O_5–x_), plausibly associated with grain-boundary oxygen vacancies generated during the high-temperature calcination employed in manufacturing the commercial material. No equivalent component is detected in the Nb75-180 spectrum, indicating that the low-temperature pressureless microwave synthesis preserves a uniform Nb^5+^ oxidation state across the surface.

**Figure 7:**
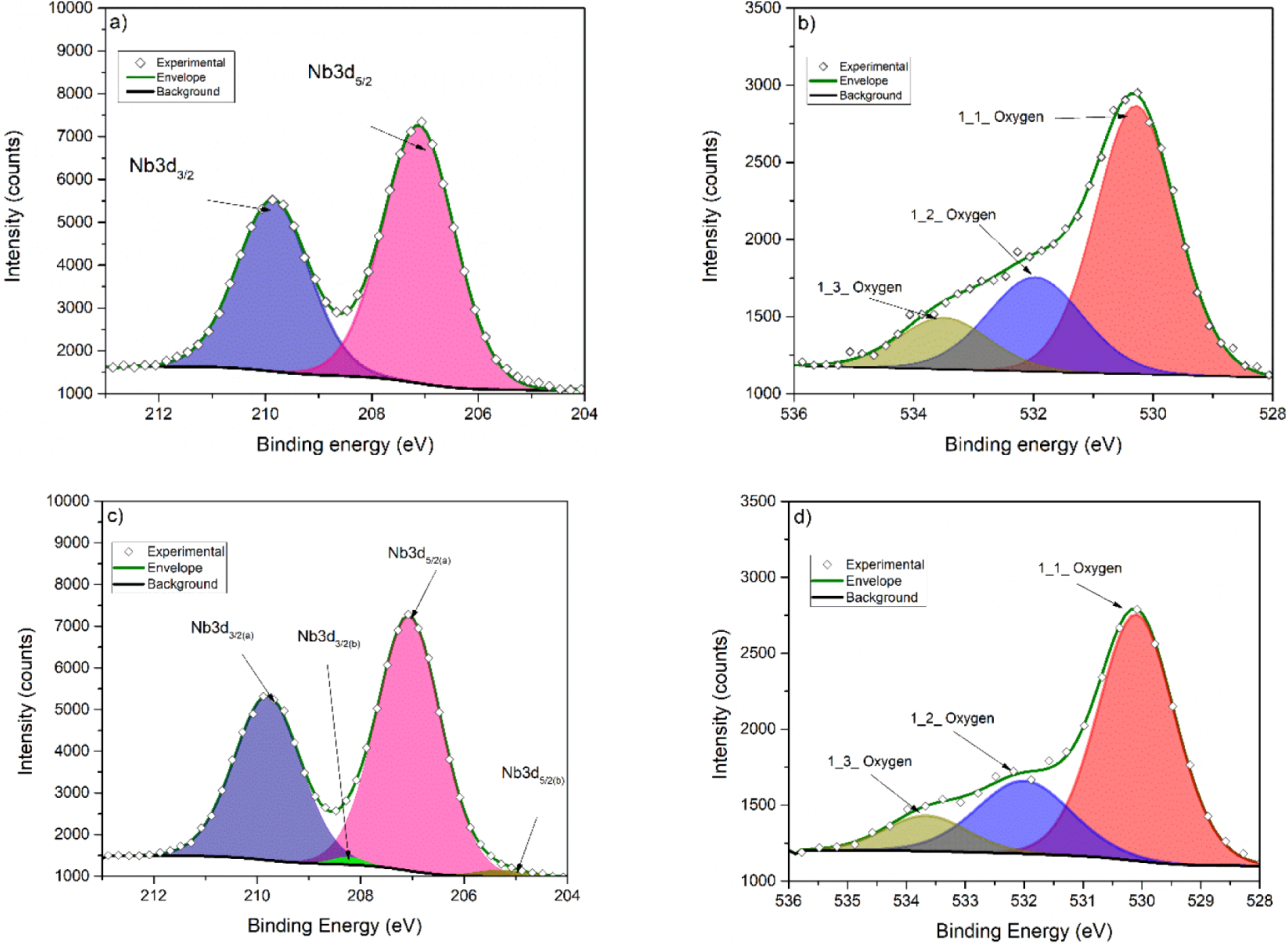
High-resolution XPS spectra of (a) Nb 3d and (b) O 1s regions for amorphous Nb75-180, and (c) Nb 3d and (d) O 1s regions for crystalline commercial Nb_2_O_5_. Panels (a) and (c) show the Nb^5+^ oxidation state with characteristic doublets at 207.1 and 209.8 eV; panel (c) additionally reveals a minor secondary doublet (∼205.1 and 208.2 eV) attributed to substoichiometric niobium sites (Nb_2_O_5–x_), which are absent in (a). Panels (b) and (d) present the O 1s region deconvoluted into three components: lattice oxygen (530.30 eV), surface hydroxyl groups (531.86–532.06 eV), and adsorbed species (533.16–533.67 eV), revealing higher surface hydroxyl content in amorphous Nb75-180 relative to crystalline commercial Nb_2_O_5_.

Concerning the Nb75-180 O 1s chemical states, presented in Figure 7b, the O1_1 at 530.30 eV, representing the dominant contribution to the O 1s envelope, unambiguously represents the lattice oxygen within the Nb_2_O_5_ structure. This assignment is consistent with the dominant O–Nb bonding environment of the Nb_2_O_5_·nH_2_O network, even in the absence of long-range crystalline order.^26^ The presence of an additional peak at 531.86 eV, O1_2, indicates that not all oxygen atoms are part of the rigid oxide lattice. This component is most reasonably assigned to surface hydroxyl groups.^27^ Furthermore, the third component at 533.16 eV, O1_3, is assigned to the adsorbed oxygen species. This peak is typically associated with weakly bound molecular species, such as adsorbed water. Commercial Nb_2_O_5_ O 1s chemical states, shown in Figure 7d, were characterized by a moderate increase in O1_2 binding energy to 532.06 eV alongside a more substantial elevation of O1_3 to 533.67 eV.

This spectroscopic evolution is attributed to the emergence of distinct surface oxygen environments on the well-defined crystalline architecture of the commercial material, in which thermal treatment during manufacturing promotes dehydroxylation and condensation of surface OH groups, as independently evidenced by the markedly attenuated ν­(O–H) and δ­(H–O–H) bands in the FTIR spectrum. The thermally induced upward displacement of the O1_2 peak indicates the establishment of reorganized oxygen coordination geometries possessing greater binding energies relative to the pristine hydroxyl-terminated surfaces. Simultaneously, the marked elevation in O1_3 binding energy signifies either the persistence or chemical transformation of strongly chemisorbed species (likely encompassing carbonate moieties or firmly coordinated water molecules) which develop enhanced binding interactions with the thermally reconstructed crystalline interface.

### 3.7 Scanning Electron Microscopy (SEM)

The scanning electron microscopy (SEM) analysis presented in Figure 8a,b reveals distinct morphological differences between the Nb75-180 sample and commercial Nb_2_O_5_. The Nb75-180 material exhibits a grape-like agglomerated morphology consisting of loosely aggregated primary particles with minimal interparticle consolidation. Conversely, the commercial Nb_2_O_5_ displays a well-defined, stratified architecture characterized by a densely consolidated monolithic matrix with surface-adsorbed or partially embedded fine particulate debris. Figure 8c,d shows an overlay of the SEM micrograph with the particle perimeters obtained by pixel-wise segmentation and connected-component labeling, where closed boundaries were delineated through isocontour-based contour tracing. This procedure enabled the unambiguous identification of individual particles, yielding 1070 detected particles for the Nb75-180 sample and 438 particles for the commercial Nb_2_O_5_.

**Figure 8:**
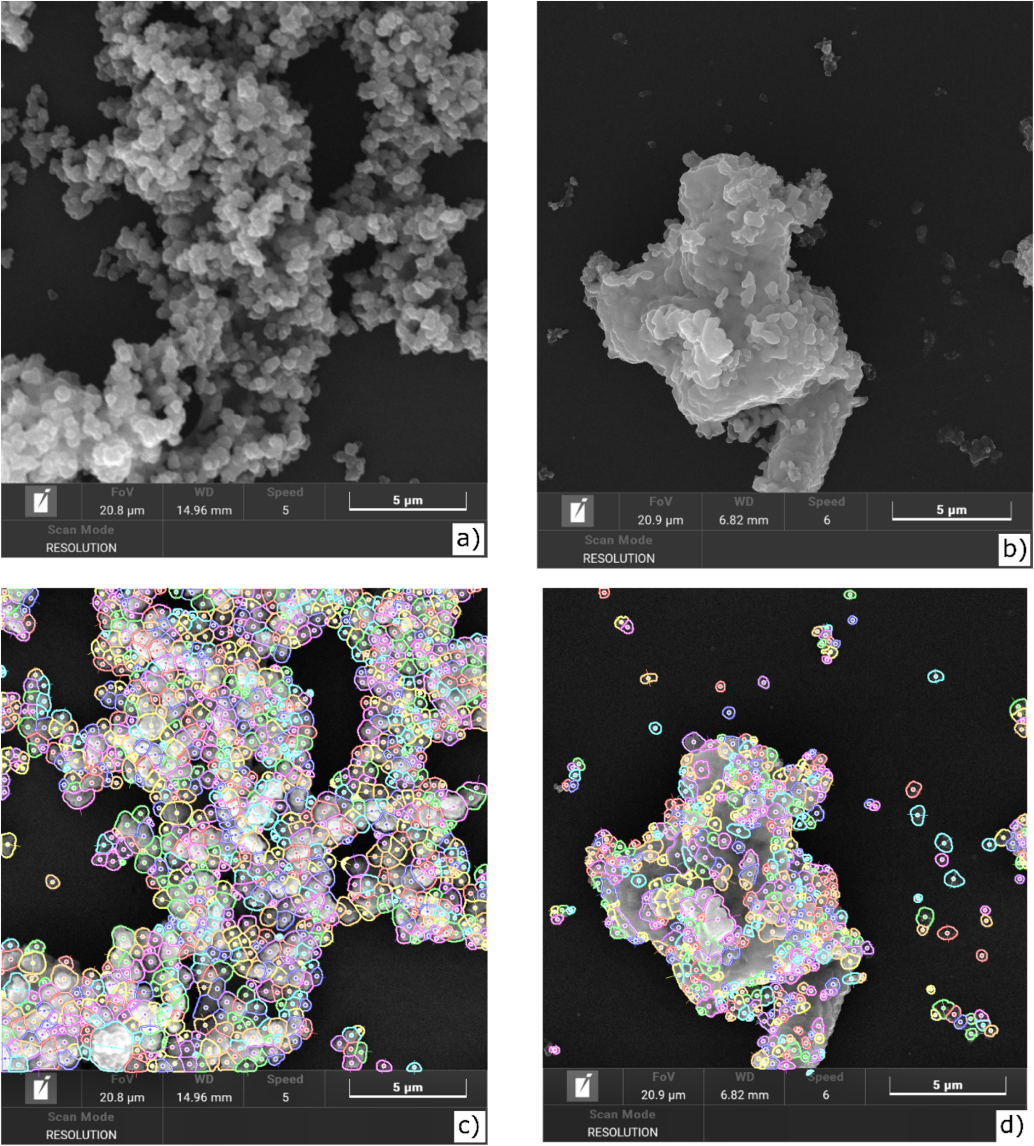
SEM micrographs of Nb75-180 and commercial Nb_2_O_5_. (a,b) Grape-like agglomerates in Nb75-180 and a dense stratified structure in the commercial sample. (c,d) Segmentation-derived particle perimeters identifying 1070 and 438 particles, respectively.

The particle size distributions’ diameters of the two specimens, depicted in Figure 9a,b, reveals fundamentally different growth regimes and levels of kinetic control. The MAPHS-derived Nb75-180 sample exhibits a well-defined and narrow size distribution, with a mean particle diameter of 5.39 × 10^2^ ± 1.75 × 10^2^ nm (σ,μ) and a median value of 5.22 × 10^2^ nm. The moderate coefficient of variation of approximately 32% and the moderate positive skewness indicate a population dominated by uniformly sized particles with only a limited fraction of larger entities, as demonstrated by

3 $$\mathrm{CV}=\frac{\sigma }{\mu }\to 32.43\%$$

**Figure 9:**
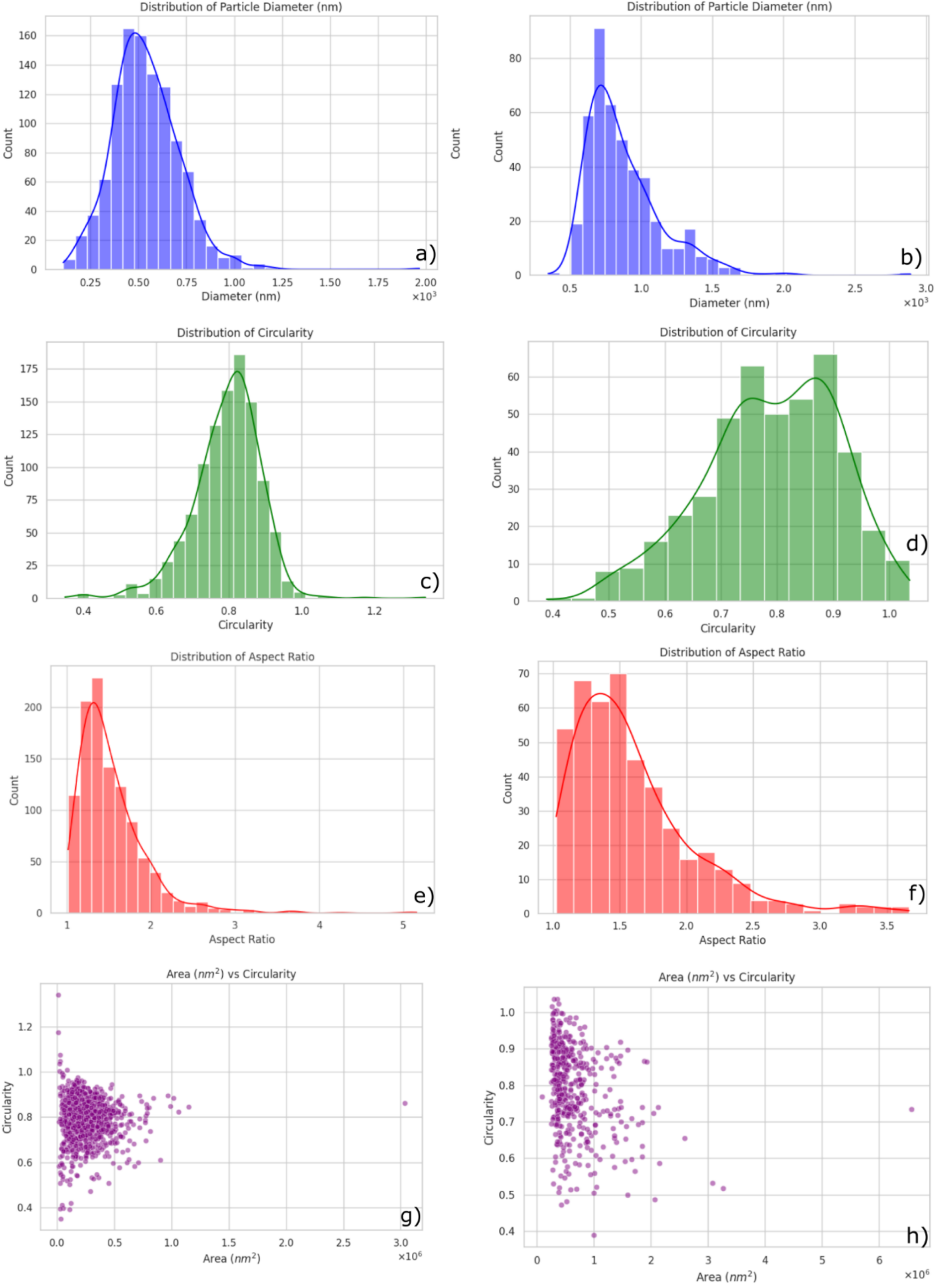
Statistical morphological analysis of Nb75-180 (left column: a, c, e, g) and commercial Nb_2_O_5_ (right column: b, d, f, h). Panels (a) and (b) show the particle diameter distributions (nm), evidencing narrow and broad growth regimes, respectively. Panels (c) and (d) present circularity histograms, highlighting a uniform shape distribution for Nb75-180 and a bimodal distribution for the commercial sample. Panels (e) and (f) display the aspect ratio distributions, indicating the degree of particle elongation. Panels (g) and (h) show scatter plots of projected area (nm^2^) versus circularity, illustrating the extent of particle aggregation and secondary cluster formation.

Such behavior is consistent with homogeneous nucleation under rapid and volumetric microwave heating, where energy is delivered uniformly throughout the reaction medium.^15^ Under these conditions, nucleation is favored over growth, and Ostwald ripening is strongly suppressed during the 180 min synthesis period.^28,29^ The strong positive skewness, as demonstrated in eq 4 and high kurtosis in eq 5, reflect the presence of a substantial tail toward larger particle sizes, characteristic of precipitation–calcination routes carried out under less stringent kinetic constraints, where prolonged thermal exposure enhances growth and coarsening.

4 $$G1=\frac{n}{\left(n-1\right)\left(n-2\right)}\overset{n}{\underset{i=1}{\sum }}{\left(\frac{xi-\mathrm{x̅}}{\delta }\right)}^{3}\to 0.878$$

where

*n*: number of particles (1070).

*x_i_*, diameter of an individual particle.

*x̅*: mean diameter of the particle population (539.02 nm).

δ: standard deviation (174.80 nm).

5 $$K=\left[\frac{n\left(n+1\right)}{\left(n-1\right)\left(n-2\right)\left(n-3\right)}\overset{n}{\underset{i=1}{\sum }}\left(\frac{xi-\mathrm{x̅}}{\delta }\right)\right]-\frac{3{\left(n-1\right)}^{2}}{\left(n-2\right)\left(n-3\right)}\to 4.26$$

where

*n*: number of particles (1070).

*x_i_ – x̅*: deviation of each particle diameter from the mean diameter.

δ, standard deviation of the particle size distribution.

In contrast, the commercial sample displays a markedly broader and less controlled size distribution, with a mean diameter of 8.8 × 10^2^ ± 270 nm (σ,μ), corresponding to particles that are on average 63% larger than those obtained by MAPHS. The Coefficient of variation for this sample according to the application of $CV=\frac{\sigma }{\mu }\to 32.43\%$ (eq 3) returns 30.68%, while the skewness and kurtosis (eq 4, eq 5) return 1.98 and 7.59, respectively. While both samples exhibit similar coefficients of variation (31–32%), the crystalline commercial Nb_2_O_5_ shows markedly different distribution shapes, as quantified by the skewness (1.98 vs 0.88) and kurtosis (7.59 vs 4.26) parameters. The moderate skewness and low kurtosis for the amorphous Nb75-180 sample reflect relatively uniform nucleation and growth kinetics during rapid precipitation, where stochastic variations in local supersaturation create some size heterogeneity, but the absence of thermodynamically driven crystal growth prevents the formation of extreme outliers. In contrast, the high skewness and kurtosis of the commercial crystalline sample are characteristic of complex multistep processes including crystal nucleation, growth, Ostwald ripening, and phase transformations between T and TT polymorphs. The pronounced tail toward larger particles (high skewness) suggests that certain crystallites experience preferential growth conditions or extended growth times, while the heavy tails at both extremes (high kurtosis) indicate the presence of distinct particle subpopulations. Despite similarities in average circularity between the two materials, their morphological distributions differ substantially as demonstrated by Figure 9c,d. The Nb75-180 specimen presents a high degree of morphological uniformity, with a mean circularity of 0.794 ± 0.094 (Figure 9c) and an aspect ratio of 1.53 ± 0.42 (Figure 9e). The particles are predominantly quasi-spherical with slight elongation, which can be attributed to isotropic growth within an amorphous matrix. During microwave-assisted synthesis, local electromagnetic field effects and transient concentration gradients may induce mild preferential elongation, yet these effects do not compromise the overall homogeneity of the particle population. The commercial material, although exhibiting a comparable mean circularity of 0.79 ± 0.12 (Figure 9d) and a similar average aspect ratio of 1.60 ± 0.46 (Figure 9f), shows a much broader dispersion of shapes, including a significant fraction of highly elongated particles with aspect ratios exceeding 2.0. The resulting bimodal circularity distribution, depicted in Figure 9c,d with distinct maxima at approximately 0.75 and 0.87, thus serves as a clear morphological signature of phase coexistence. Aggregation behavior further accentuates the contrast between the two specimens. The Nb75-180 sample shows limited aggregation, with the majority of particles exhibiting projected areas below 0.5 μm^2^ while maintaining high circularity values above 0.6. Even the relatively larger entities, exceeding 1.0 μm^2^, preserve substantial circularity, indicating that they correspond to individual primary particles rather than to agglomerates formed by coalescence. This behavior reflects the kinetically arrested nature of the low-temperature MAPHS process at 75 °C, in which particle surfaces remain insufficiently activated to enable neck formation or sintering. In sharp contrast, the commercial sample displays pronounced aggregation, as evidenced by extreme kurtosis in the area distribution and the presence of clusters reaching areas as large as 6.5 μm^2^. Particles in the 0.7–1.2 μm range with high aspect ratios can be identified as primary crystallites, whereas larger features exceeding 2.0 μm with reduced circularity are indicative of secondary aggregates. This hierarchical microstructure is characteristic of high-temperature calcination treatments, typically in the 400–600 °C range for the TT-to-T phase transformation, where enhanced atomic mobility promotes solid-state diffusion, interparticle necking, and the formation of hard agglomerates.

### 3.8 NaBH_4_ Methanolysis over Synthesized Nb_2_O_5_·nH_2_O Catalyst

After the synthesis and characterization steps, the catalytic performance of the Nb_2_O_5_·nH_2_O catalyst synthesized by the microwave-assisted hydrothermal method was investigated. Figure 10 depicts the hydrogen released as a function of time, through the methanolysis of NaBH_4_, in the absence and in the presence of synthesized Nb_2_O_5_·nH_2_O acting as a catalyst.

**Figure 10:**
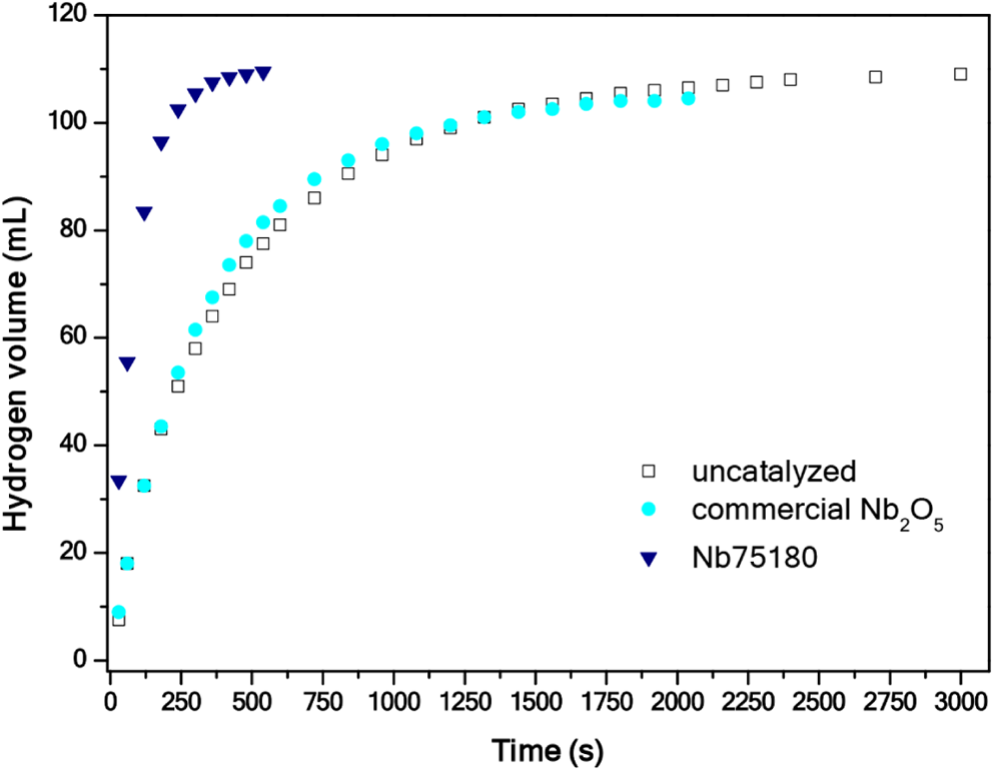
Evolution of hydrogen volume over time for the methanolysis of NaBH_4_ using Nb75-180 catalyst, commercial Nb_2_O_5_ catalyst, and a blank (in the absence of a catalyst). Reaction conditions: 50 mg Nb75-180 catalyst, 62.5 mM NaBH_4_ solution, 25 °C.

For comparative purposes, the result of the assay performed with the commercial Nb_2_O_5_ catalyst is shown. It can be observed that hydrogen production vs time was higher in the case of Nb75-180 catalyzed reaction. Without a catalyst, the reaction proceeds at a slower rate. The commercial catalyst displayed minimal activity, performing similarly to the blank (noncatalyzed) reaction. Conversely, the Nb75-180 catalyst is more active than its commercial counterpart. The time required to achieve the expected hydrogen volume becomes shorter with the introduction of the Nb75-180 catalyst. The rate of hydrogen production in the self-methanolysis reaction was 10.9 mL H_2_·min^–1^, whereas that of the Nb75-180-catalyzed reaction was calculated as 32.6 mL H_2_·min^–1^. The HGR (hydrogen generation rate) for Nb75-180 catalyst was recorded as 652.0 mL H_2_·min^–1^·g_cat_^–1^ and for the commercial Nb_2_O_5_ catalyst as 252.1 mL H_2_·min^–1^·g_cat_^–1^, under the experimental conditions associated with Figure 10, i.e., with 50 mg of catalyst. The reaction is about two and a half times faster with Nb75-180. The enhanced Nb75-180 catalytic activity can be interpreted in light of its larger specific surface area. Nevertheless, this may not be considered the main driver of its superior performance toward NaBH_4_ methanolysis. Based on the characterization analyses previously discussed, it can be inferred that the density and nature of the active sites (the Brønsted acidity and the presence of moderate acid sites) on the catalyst surface should play the main role within the catalytic scene, given that the reaction is known as an acid-catalyzed reaction.^30^ In the NaBH_4_ methanolysis reaction, borohydride ions (BH_4_^–^) and CH_3_OH reactants can be adsorbed on the catalyst surface. In this process, the acidic properties of the catalyst directly influence the adsorption of reactant molecules and the cleavage mechanism of B–H and O–H bonds for hydrogen release. Nb_2_O_5_·nH_2_O is a well-known heterogeneous acid catalyst, featuring both Brønsted and Lewis acid sites on its surface.^31^ Brønsted acid sites, originating from the hydroxyl groups on the Nb75-180 surface, act as proton donors, facilitating the protonation of the borohydride anion (BH_4_^–^). This weakens the boron–hydrogen bond, allowing hydrogen to be released more easily. In turn, the Lewis acid sites, originating from niobium atoms, coordinate with the BH_4_^–^ or with methanol molecules, increasing bond polarization and facilitating the nucleophilic attack of methanol on the boron atom, resulting in the hydrogen release. This sequential process ultimately leads to the formation of the NaB­(OCH_3_)_4_ byproduct.^31,32^ In stark contrast to the behavior of the Nb75-180 catalyst, the very low activity exhibited by the Nb_2_O_5_ commercial sample may be related not only to its small specific surface area but also to the predominant presence of moderate and strong acid sites. Very strong Lewis acid sites (likely present in the commercial catalyst) bind too strongly to the borohydride anion and methanol molecules, leading to catalyst poisoning and hindering the reaction.

Next, the impact of some reaction parameters on the hydrogen production rate from NaBH_4_ methanolysis promoted by the Nb75-180 catalyst was evaluated. In this regard, the influence of the catalyst amount was studied using 12.5 mg, 25 mg, and 50 mg of the Nb75-180 catalyst. The hydrogen generation rate (HGR) decreased with the increase in catalyst mass. When the catalyst amount increased up to 50 mg, the hydrogen generation rate dropped, as shown in the inset of Figure 11. In certain cases, HGR would increase with the catalyst amount, since more active sites would be available. However, an opposite trend was observed. This behavior is because the adsorption of reactant molecules (BH_4_^–^ and/or CH_3_OH species) on the catalyst surface is the rate-determining step in the methanolysis reaction, which could likely be limited by mass transfer. If there is a lot of catalyst present in the medium, the methanolysis reaction produces hydrogen gas very quickly during the initial period of the process (as can be carefully observed from the slopes of the initial branches of the curves in Figure 11a. The massive generation of gas bubbles on the surface of the solid can shield the catalyst, making it difficult for the reactant molecules to reach the active sites. Furthermore, the use of high catalyst quantities can make it less accessible due to the formation of agglomerates that hinders its dispersion in the medium, reducing the active surface area effectively exposed to the reactants. It should also be taken into account that the mass of the catalyst is present in the denominator of the equation used in the HGR calculation. Thus, if part of the catalyst surface is devoid of adsorbed species due to a large amount of catalyst relative to the reactants, this leads to a decrease in HGR values.^29,33^ Figure 11b shows the effect of catalyst mass loading on the absolute hydrogen production rate (mL H_2_·min^–1^). Under the experimental conditions employed for the 62.5 mM NaBH_4_ solution, the linear behavior, with a slope of 0.40, suggests that only 12.5 mg of material is required for subsequent catalytic tests. Importantly, the decrease in HGR is a consequence of per-mass normalization for the NaBH_4_ concentration solution applied, combined with mass-transfer and agglomeration effects at higher loadings, and not a loss of intrinsic catalytic activity. Therefore, the following tests were conducted with 12.5 mg of the catalyst.

**Figure 11:**
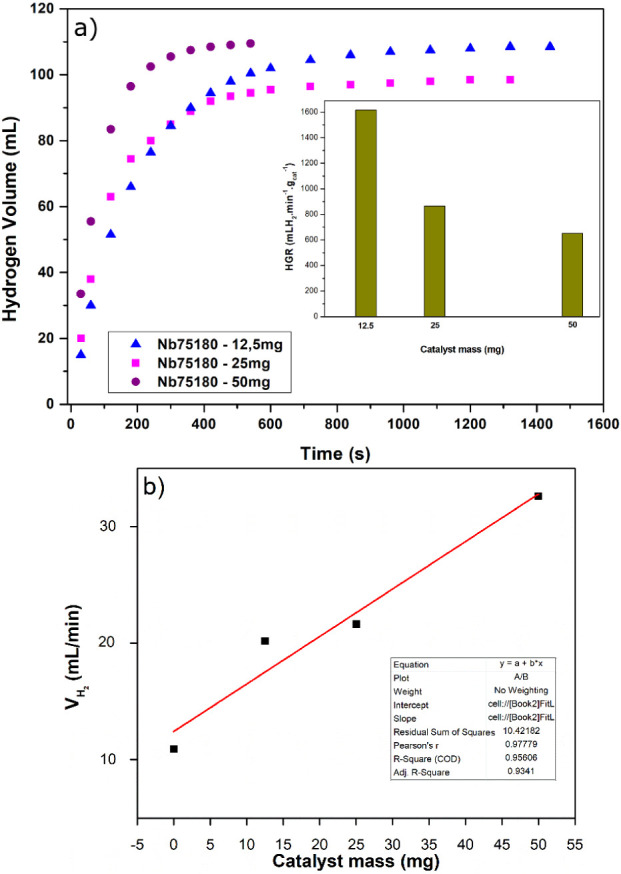
(a) Hydrogen production from the methanolysis of NaBH_4_ in the presence of different masses of Nb75-180 catalyst. Inset: hydrogen generation rate (HGR) as a function of catalyst mass. Reaction conditions: 12.5, 25, and 50 mg Nb75-180 catalyst, respectively; 62.5 mM NaBH_4_ solution; 25 °C. (b) Volume of the produced hydrogen vs catalyst amount.

Hydrogen production efficiency at five different NaBH_4_ concentrations (31.25 mM, 62.5 mM, 125 mM, 250 mM, and 500 mM) was evaluated. The solution concentration was adjusted by varying the NaBH_4_ mass while keeping the methanol volume constant. The graph of the generated hydrogen volume as a function of time is depicted in Figure 12a. An increase in hydrogen production volume can be seen as the substrate concentration increases because of NaBH_4_ methanolysis reaction stoichiometry. While the HGR obtained from the methanolysis of the 31.25 mM NaBH_4_ solution was 1243.9 mL H_2_·min^–1^·g_cat_^–1^, the HGR value obtained with 500 mM NaBH_4_ solution was found to be 22781.7 mL H_2_·min^–1^·g_cat_^–1^, that is approximately 18 times higher. The linear plot of ln­(HGR) versus ln­[NaBH_4_] yields a slope of 1.062, Figure 12b (inset), demonstrating a first-order dependence of the reaction rate on NaBH_4_ concentration, which is consistent with the integrated first-order rate equation, ln­[A] = −kt + ln­[A]_0_. This kinetic behavior indicates that the methanolysis of NaBH_4_ over the Nb_2_O_5_ catalyst proceeds via a rate-determining step that is directly proportional to the NaBH_4_ concentration, as confirmed by the excellent agreement between the experimental data and classical first-order reaction kinetics.^34,35^

**Figure 12:**
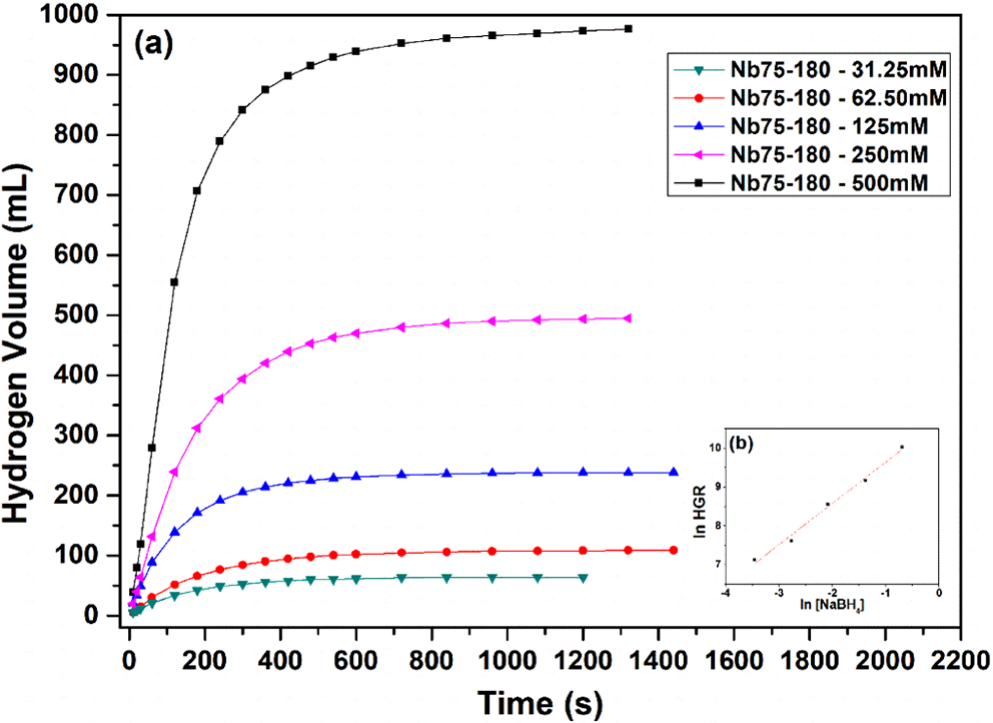
a) Hydrogen production from the methanolysis of NaBH_4_ in the presence of different NaBH_4_ concentrations and b) ln­(HGR) vs ln­(NaBH_4_ concentration). Reaction conditions: 12.5 mg Nb75-180 catalyst; 31.25 mM, 62.5 mM, 125 mM, 250 mM, and 500 mM NaBH_4_ solution, respectively; 25 °C.

The influence of methanol volume on hydrogen production from the catalytic methanolysis of sodium borohydride is an important factor, albeit sometimes secondary compared to temperature or the amount of NaBH_4_. To investigate the effect of methanol volume, experiments varying the methanol volume from 10 to 50 mL, while keeping the other parameters constant, were carried out, as shown in Figure 13. The HGR was determined for all methanol volumes to assess their effect on the reaction’s progress (Figure 13, inset). From the results, it is clear that methanol volume has an impact on hydrogen generation. As the volume of methanol increases, the HGR decreases. This decline in HGR occurs because the excess methanol dilutes the reaction mixture. Reducing the reactant and catalyst concentrations hinders the interaction between them, thereby limiting the hydrogen production efficiency.^36^

**Figure 13:**
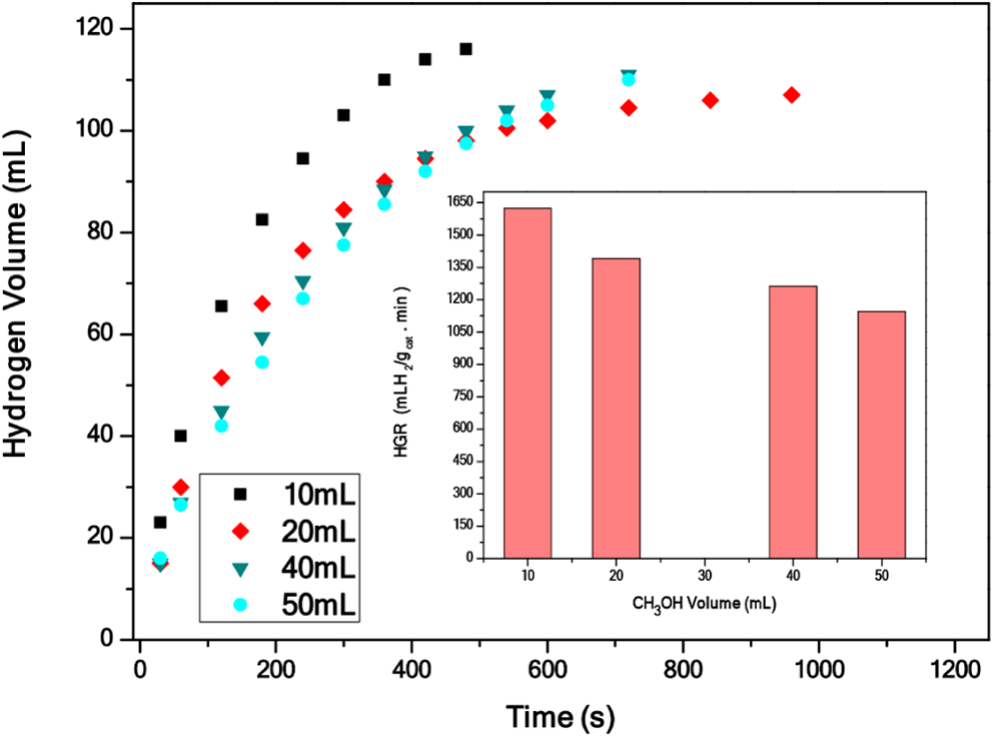
Hydrogen production from the methanolysis of NaBH_4_ in the presence of different CH_3_OH volumes. Inset: hydrogen generation rate (HGR) as a function of CH_3_OH volume. Reaction conditions: 12.5 mg Nb75-180 catalyst; 10 mL, 20 mL, 40 mL, and 50 mL of CH_3_OH, respectively; 62.5 mM NaBH_4_ solution; 25 °C.

Figure 14a shows the effect of reaction temperature on hydrogen production from NaBH_4_ methanolysis catalyzed by the Nb75-180 sample, while keeping the amount of catalyst and the NaBH_4_ concentration constant. The rate of hydrogen production increased significantly with the increase in temperature, reaching the maximum HGR of 8184.4 mL H_2_·min^–1^·g_cat_^–1^ at 55 °C. The activation energy (E_a_) of the reaction was determined through the Arrhenius law. The Arrhenius plot, which is ln­(k) versus 1/T, is depicted in Figure 14b­(inset). The E_a_ value calculated was 21.95 kJ/mol.

**Figure 14:**
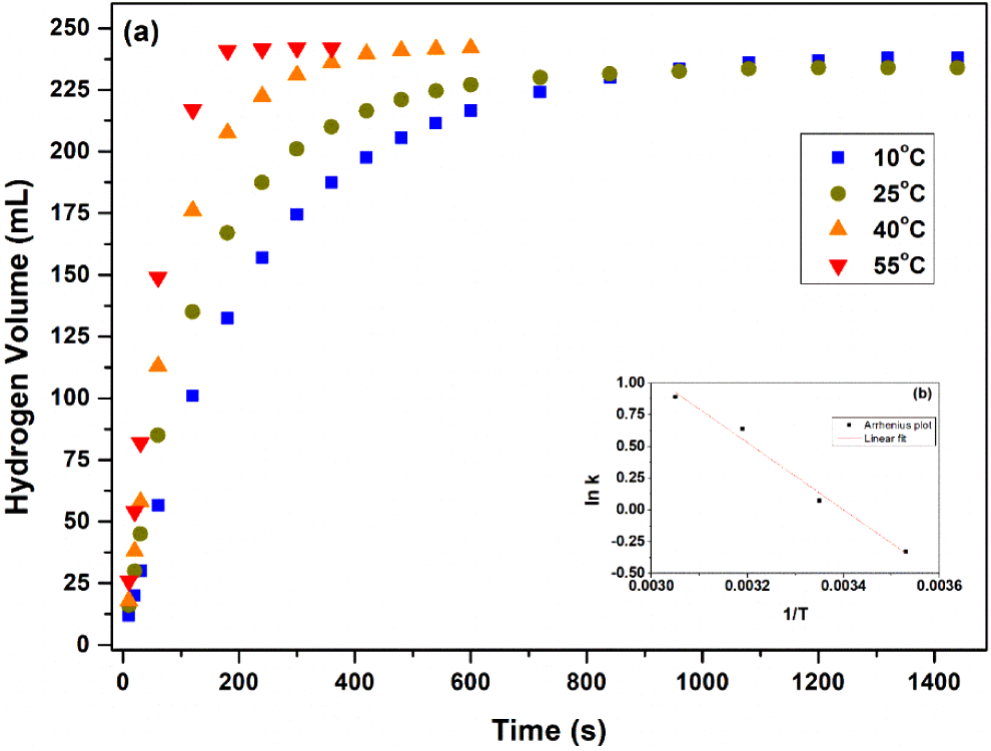
a) Hydrogen production curves from the methanolysis of NaBH_4_ at different temperatures. Reaction conditions: 12.5 mg Nb75-180 catalyst; 125 mM NaBH_4_ solution; 10 °C, 25 °C, 40 °C, and 55 °C, respectively; b) Arrhenius equation graph to calculate the activation energy for the NaBH_4_ methanolysis reaction.

For practical applications, recyclability is a desirable feature of a high-performing catalyst. It was investigated how many times the synthesized Nb75-180 could be used before it began to lose its ability to catalyze the methanolysis reaction. Figure 15 depicts the time-dependent hydrogen production curves and HGR values (inset) for the Nb75-180 catalyst in three consecutive cycles. The reaction rate slightly decreases after the first cycle, as can be seen by the changes in the profiles of the hydrogen production curves over time. The time required to achieve the expected hydrogen volume became longer with consecutive cycles. However, the HGR value varies very little over the three cycles. During the recovery of the catalyst for reuse, there was a loss of its mass; consequently, the catalyst amounts used in the subsequent tests were lower. This quantity appears in the denominator of the equation used to calculate HGR. In this way, after the third cycle, the catalytic efficiency was practically maintained without significant HGR loss. Based on these results, it can be said that the synthesized Nb75-180 catalyst has very good stability, and it can be used in consecutive cycles in the sodium borohydride methanolysis reaction.

**Figure 15:**
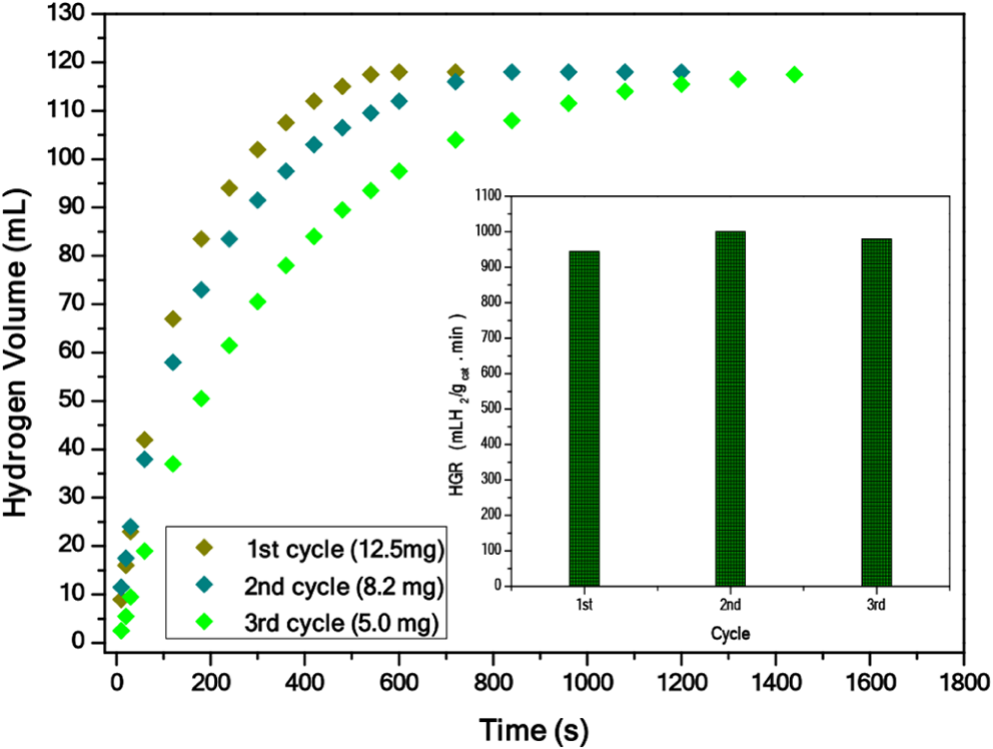
Reusability performance expressed by hydrogen evolution curves from the methanolysis of NaBH_4_ catalyzed by Nb75-180. Inset: hydrogen generation rate (HGR) as a function of consecutive cycles. Reaction conditions: 12.5, 8.2, and 5.0 mg Nb75-180 catalyst, respectively; 62.5 mM NaBH_4_ solution; 25 °C.

With the aim of positioning the Nb75-180 catalyst’s outstanding performance, a brief compilation of the catalytic performances available in the literature is made, and the corresponding results are tabulated in Table 1. The activation energy of the Nb75-180 catalyst for the methanolysis of NaBH_4_ was comparable to or lower, and the HGR was also comparable to or, in some cases, higher than that of various previously reported catalysts, including noble-metal-based catalysts. In addition, from Table 1, it can be seen that the reported catalysts showed activity loss at different levels. Such a process was not observed in this study. Therefore, the stability presented by the Nb75-180 catalyst is a point that deserves to be highlighted.

**Table 1:** Comparison of the Catalytic Performances between Synthesized Nb75-180 Catalyst and Different Catalysts for NaBH_4_ Methanolysis Previously Reported

Catalyst	HGR^a^ (mL·min^–1^·g_cat_^–1^)	E_a_ (kJ mol^–1^)	Reusability (cycles)	Reference
Pd–Co_3_O_4_/SN-CNS-600	20158	14.52	10 (no loss)	^37^
Ru–Co/CNTs	21190	34.35	5 (25% loss)	^38^
BiOCl	42740	43.5	5 (no loss)	^39^
Co–P/CNTs-Ni foam	2430	49.94	8 (26% loss)	^39^
Fe_3_O_4_@SiO_2_@NH_3_^+^Cl^®^	13188	24.4	5 (40% loss)	^40^
Natural lignin	20425	19.5	5 (34% loss)	^41^
MGCell-PEI^+^	3463	21.7	5 (22% loss)	^42^
Cell-EPC-DETA-HCl	3215	34.4	5 (35% loss)	^43^
g-C_3_N_4_–TiO_2_–P	14750	36.17	5 (43% loss)	^44^
Ru/NiO-Ni foam	6000	42.33	10 (20% loss)	^45^
Nb75-180	22782	21.95	3 (no loss)	Present work

a Reaction temperature range = 25–30 °C.

## 4 Conclusions

This investigation establishes that amorphous Nb_2_O_5_·nH_2_O synthesized via pressureless microwave-assisted hydrothermal processing represents a highly efficient, stable, and sustainable catalyst for hydrogen evolution from sodium borohydride methanolysis. The open-vessel microwave methodology facilitates precise kinetic control under sub-boiling conditions, yielding a nanostructured material characterized by high specific surface area, accessible microporosity, and a surface chemistry enriched in weak-to-moderate acidic sites. These physicochemical attributes collectively govern the exceptional catalytic performance, exemplified by a hydrogen generation rate of 22781.7 mL·min^–1^·g^–1^ at 25 °C and an apparent activation energy of 21.95 kJ·mol^–1^, metrics that surpass numerous noble-metal-based catalysts while exhibiting excellent cyclability and operational stability. Complementing conventional characterization techniques, the integration of machine learning-assisted analysis of scanning electron microscopy data provides a quantitative, statistically rigorous framework for elucidating catalyst morphology and its genesis. Automated particle segmentation coupled with multivariate feature extraction revealed a narrow particle size distribution, moderate skewness, and low kurtosis for the microwave-synthesized Nb_2_O_5_, features consistent with homogeneous nucleation and kinetically arrested growth under rapid, uniform heating. Conversely, the crystalline commercial Nb_2_O_5_ exhibited pronounced positive skewness, elevated kurtosis, and substantial morphological heterogeneity-signatures of complex, multistep growth processes encompassing Ostwald ripening, grain boundary migration, and phase coexistence.^18^ These data-driven morphological descriptors furnish direct evidence that controlled microwave-induced nucleation effectively suppresses excessive particle coarsening and agglomeration, thereby maximizing catalytically active surface area. Importantly, the machine learning-derived morphological metrics demonstrate robust correlation with catalytic performance, thereby establishing a quantitative structure–morphology–activity relationship. The narrow size distribution and minimal agglomeration characteristic of the amorphous Nb_2_O_5_ enhance reactant diffusion pathways and promote efficient exploitation of surface acidic sites, whereas the morphological heterogeneity inherent to the crystalline counterpart diminishes catalytic efficacy. This quantitative linkage, enabled through advanced computational methods, substantially deepens mechanistic insight into how synthesis parameters govern catalytic behavior beyond the limitations of conventional metrics such as mean particle size or BET surface area.

## References

[1] Zainal B. S., Ker P. J., Mohamed H., Ong H. C., Fattah I. M. R., Rahman S. M. A., Nghiem L. D., Mahlia T. M. I. (2024). Recent Advancement
and Assessment of Green Hydrogen Production Technologies. Renewable Sustainable Energy Rev..

[2] Xu D., Zhang Y., Guo Q. (2022). Research Progress on Catalysts for
Hydrogen Generation through Sodium Borohydride Alcoholysis. Int. J. Hydrogen Energy.

[3] Liu B. H., Li Z. P. (2009). A Review: Hydrogen
Generation from Borohydride Hydrolysis Reaction. J. Power Sources.

[4] Bekirogullari M. (2024). Synthesis
of Waste Eggshell-Derived Au/Co/Zn/Eggshell Nanocomposites for Efficient
Hydrogen Production from NaBH4Methanolysis. Int. J. Hydrogen Energy.

[5] Santos D. M. F., Sequeira C. A. C. (2011). Sodium Borohydride
as a Fuel for
the Future. Renewable Sustainable Energy Rev..

[6] Dragan M. (2022). Hydrogen Storage
in Complex Metal Hydrides NaBH4: Hydrolysis Reaction and Experimental
Strategies. Catalysts.

[7] Akin M. B. (2024). Hydrogen
Generation from Methanolysis of Sodium Borohydride Using Boric Acid
as a Catalyst. Energy Sources, Part A.

[8] Nakajima K., Baba Y., Noma R., Kitano M., Kondo J. N., Hayashi S., Hara M. (2011). Nb_2_ O_5_ ·nH_2_ O as a Heterogeneous Catalyst with Water-Tolerant Lewis Acid
Sites. J. Am. Chem. Soc..

[9] Mendes F. M. T., Perez C. A., Soares R. R., Noronha F. B., Schmal M. (2003). Ammonium Complex
of Niobium as a Precursor for the Preparation of Nb2O5/Al2O3 Catalysts. Catal. Today.

[10] Zhao Y., Zhou X., Ye L., Chi Edman Tsang S. (2012). Nanostructured
Nb_2_ O_5_ Catalysts. Nano
Rev..

[11] Li H., Chen H., Chen Y., Bai G., Zhang M., Xie S., Zhuo K. (2021). Self-Branched Nb_2_ O_5_ Nanoarrays
as “Electron-Ion Reservoirs” to Enhance the Conversion
of Polysulfides in Flexible Li–S Batteries. Inorg. Chem. Front..

[12] Nowak I., Ziolek M. (1999). Niobium Compounds:
Preparation, Characterization, and
Application in Heterogeneous Catalysis. Chem.
Rev..

[13] Carniti P., Gervasini A., Biella S., Auroux A. (2005). Intrinsic and Effective
Acidity Study of Niobic Acid and Niobium Phosphate by a Multitechnique
Approach. Chem. Mater..

[14] Rangel E. M., Riemke F. C., Ücker C. L., Raubach C. W., Adebayo M. A., Machado Machado F. (2022). Photodegradation
of Acid Yellow 23 BY Nb2O5 Supported
on Eco-Friendly Glass Foams. J. Clean. Prod..

[15] Riemke F. C., Ücker C. L., Carreño N. L.
V., da Silva
Cava S., Teixeira M. P., Fajardo H. V., Taylor J. G., da Silva M. J., Batalha D. C., Raubach C. W. (2022). Influence of Nb2O5 Grown on SrTiO3
Nanoseeds in the Catalytic Oxidation of Thioanisole. Mater. Chem. Phys..

[16] Silva R. M., Noremberg B. S., Marins N. H., Milne J., Zhitomirsky I., Carreño N. L. (2019). Microwave-Assisted Hydrothermal Synthesis and Electrochemical
Characterization of Niobium Pentoxide/Carbon Nanotubes Composites. J. Mater. Res..

[17] Stuerga, D. Microwave-Material Interactions and Dielectric Properties, Key Ingredients for Mastery of Chemical Microwave Processes Microwaves in Organic Synthesis Wiley 2006 2 1–61 10.1002/9783527619559.ch1

[18] Ücker C. L., Goetzke V., Riemke F. C., Vitale M. L., De Andrade L. R. Q., Ücker M. D., Moreira E. C., Moreira M. L., Raubach C. W., Cava S. S. (2021). Multi-Photonic
Behavior of Nb2O5
and Its Correlation with Synthetic Methods. J. Mater. Sci..

[19] Costa G. P., Gonçalves A. H. A., Viana L. A. V., Soares J. C. S., Passos F. B., Mendes F. M. T., Gaspar A. B. (2021). Role of ZnNb2O6
in ZnO-Promoted Amorphous-Nb2O5 Supported Ru Catalyst for the Partial
Hydrogenation of Benzene. Mater. Today Chem..

[20] Osuga R., Hiyoshi Y., Yokoi T., Kondo J. N. (2021). Synthesis of NaNbO3
under Non-Hydrothermal Conditions from Sodium Niobate Precursors Prepared
by Alkaline Treatment of Amorphous Nb2O5. J.
Solid State Chem..

[21] Shan Y., Zheng Z., Liu J., Yang Y., Li Z., Huang Z., Jiang D. (2017). Niobium Pentoxide: A Promising Surface-Enhanced
Raman Scattering Active Semiconductor Substrate. Npj Comput. Mater..

[22] Thommes M., Kaneko K., Neimark A. V., Olivier J. P., Rodriguez-Reinoso F., Rouquerol J., Sing K. S. W. (2015). Physisorption
of Gases, with Special
Reference to the Evaluation of Surface Area and Pore Size Distribution
(IUPAC Technical Report). Pure Appl. Chem..

[23] Zasadzinski, J. ; Albee, B. ; Bishnoi, S. ; Cao, C. ; Ciovati, G. ; Cooley, L. ; Ford, D. ; Proslier, T. Raman Spectroscopy as a Probe of Surface Oxides and Hydrides on Niobium. Proceedings Of The SRF; Thomas Jefferson National Accelerator Facility (Jefferson Lab), 2011.

[24] Skrodczky K., Antunes M. M., Han X., Santangelo S., Scholz G., Valente A. A., Pinna N., Russo P. A. (2019). Niobium
Pentoxide Nanomaterials with Distorted Structures as Efficient Acid
Catalysts. Commun. Chem..

[25] Hardcastle F. D., Wachs I. E. (1991). Determination of
Vanadium-Oxygen Bond Distances and
Bond Orders by Raman Spectroscopy. J. Phys.
Chem..

[26] Wagner, C. D. NIST X-Ray Photoelectron Spectroscopy Database. In NIST Standarad Reference Database 20; NIST, 2000.

[27] Chen Z., Ye Y., Feng X., Wang Y., Han X., Zhu Y., Wu S., Wang S., Yang W., Wang L. (2023). High-Density Frustrated
Lewis Pairs Based on Lamellar Nb2O5 for Photocatalytic Non-Oxidative
Methane Coupling. Nat. Commun..

[28] Falk G., Borlaf M., López-Muñoz M. J., Fariñas J. C., Rodrigues Neto J. B., Moreno R. (2017). Microwave-Assisted
Synthesis of Nb2O5
for Photocatalytic Application of Nanopowders and Thin Films. J. Mater. Res..

[29] Batalha D. C., Hadler Marins N., Marques R., Lenin Villarreal Carreño N., Vieira Fajardo H., da Silva M. J., Da Silva M. J. (2020). Oxidation of Terpenic
Alcohols with Hydrogen Peroxide Promoted by Nb2O5 Obtained by Microwave-Assisted
Hydrothermal Method. Mol. Catal..

[30] Sahiner N. (2017). Modified Multi-Wall
Carbon Nanotubes as Metal Free Catalyst for Application in H2 Production
from Methanolysis of NaBH4. J. Power Sources.

[31] Bousada G. M., Nogueira Da Silva V., Fernandes De Souza B., De Oliveira R. S., Machado Junior I., Da Cunha C. H. F., Astruc D., Teixeira R. R., Lopes
Moreira R. P. (2024). Niobic Acid as a Support for Microheterogeneous Nanocatalysis
of Sodium Borohydride Hydrolysis under Mild Conditions. RSC Adv..

[32] Saka C. (2022). Phosphorus
and Sulphur-Doped Microalgae Carbon as a Highly Active Metal-Free
Catalyst for Efficient Hydrogen Release in NaBH4Methanolysis. Fuel.

[33] Ristić M., Popović S., Musić S. (2004). Sol–Gel Synthesis and Characterization
of Nb2O5 Powders. Mater. Lett..

[34] Sahiner N., Sengel S. B. (2017). Various Amine Functionalized Halloysite Nanotube as
Efficient Metal Free Catalysts for H 2 Generation from Sodium Borohydride
Methanolysis. Appl. Clay Sci.

[35] Darabi R., Gu Q., Mehrizi A. A., Altuner E. E., Alhrasishawi W., Gulbagca F., Tiri R. N. E., Tekeli Y., Seyrankaya A., Kaynak I., Sen F. (2023). Synthesis,
Characterization, and
Enhanced Hydrogen Generation from NaBH4Methanolysis of Highly Dispersed
Bimetallic PdNi Nanoparticles Supported on Vulcan Carbon. Mol. Catal..

[36] Najri B. A., Saidi K. M., Kaya C., Yıldız D., Kivrak A., Kivrak H. (2025). Graphene Oxide–Graphite Composite
Catalysts for Hydrogen Evolution in Sodium Borohydride Methanolysis. ACS Appl. Nano Mater..

[37] Prabu S., Chiang K.-Y. (2023). Synergistic Effect
of Pd-Co3O4 Nanoparticles Supported
on Coffee-Derived Sulfur, Nitrogen-Codoped Hierarchical Porous Carbon
for Efficient Methanolysis of NaBH4. J. Alloys
Compd..

[38] Kong L., Yu T., Zhan J., Zhang Y., Li G., Wang H., Wang X., Cheng L., Wang F. (2020). Study on the Performance
of NaBH_4_ Using Ru-Co/CNTs Catalyst to Catalyze Alcoholysis
to Produce Hydrogen. Fullerenes, Nanotubes,
Carbon Nanostruct..

[39] Wang F., Zhang Y., Wang Y., Luo Y., Chen Y., Zhu H. (2018). Co-P Nanoparticles Supported on Dandelion-like
CNTs-Ni Foam Composite
Carrier as a Novel Catalyst for Hydrogen Generation from NaBH4Methanolysis. Int. J. Hydrogen Energy.

[40] Sahiner N., Yasar A. O., Aktas N. (2017). H2 Generation
from NaBH4Methanolysis
via Magnetic Field Sensitive Ionic Liquid Coated Silica Particles
as Catalyst. Surf. Interfaces.

[41] Ma M., Zhou W., Zhang Y., Huang Q., Hu Z., Ouyang L. (2024). Natural Lignin as Efficient
Catalyst for H2 Production
from NaBH4Methanolysis at Low Temperature. J.
Environ. Chem. Eng..

[42] Can M., Demirci S., Sunol A. K., Philippidis G., Sahiner N. (2020). Natural Celluloses as Catalysts in Dehydrogenation
of NaBH_4_ in Methanol for H_2_ Production. ACS Omega.

[43] Sahiner N., Demirci S. (2017). Natural Microgranular
Cellulose as Alternative Catalyst
to Metal Nanoparticles for H2 Production from NaBH4Methanolysis. Appl. Catal., B.

[44] Saka C. (2022). Phosphorus
Decorated G-C3N4-TiO2 Particles as Efficient Metal-Free Catalysts
for Hydrogen Release by NaBH4Methanolysis. Fuel.

[45] Wang F., Luo Y., Zhang Y., Wang Y., Zhu H. (2020). Preparation of Bush-Like
Ru/NiO-Ni Foam Catalyst and Its Performance in Hydrogen Production
from Sodium Borohydride Alcoholysis. Energy
Fuels.

